# Reliable Radiologic Skeletal Muscle Area Assessment—A Biomarker for Cancer Cachexia Diagnosis

**DOI:** 10.3390/cells15060515

**Published:** 2026-03-13

**Authors:** Sabeen Ahmed, Nathan Parker, Margaret Park, Daniel Jeong, Lauren C. Peres, Evan W. Davis, Jennifer B. Permuth, Erin M. Siegel, Matthew B. Schabath, Yasin Yilmaz, Ghulam Rasool

**Affiliations:** 1 Department of Machine Learning, H. Lee Moffitt Cancer Center and Research Institute, Tampa, FL 33612, USA; ghulam.rasool@moffitt.org; 2 Department of Electrical Engineering, University of South Florida, Tampa, FL 33620, USA; yasiny@usf.edu; 3 Department of Health Outcomes and Behavior, H. Lee Moffitt Cancer Center and Research Institute, Tampa, FL 33612, USA; nathan.parker@moffitt.org; 4 Department of GI Oncology, H. Lee Moffitt Cancer Center and Research Institute, Tampa, FL 33612, USA; margaret.park@moffitt.org (M.P.); evan.davis@moffitt.org (E.W.D.); jenny.permuth@moffitt.org (J.B.P.); 5 Department of Biostatistics and Bioinformatics, H. Lee Moffitt Cancer Center and Research Institute, Tampa, FL 33612, USA; 6 Diagnostic Imaging and Interventional Radiology, H. Lee Moffitt Cancer Center and Research Institute, Tampa, FL 33612, USA; daniel.jeong@moffitt.org; 7 Department of Cancer Epidemiology, H. Lee Moffitt Cancer Center and Research Institute, Tampa, FL 33612, USA; lauren.peres@moffitt.org (L.C.P.); erin.siegel@nih.gov (E.M.S.); matthew.schabath@moffitt.org (M.B.S.)

**Keywords:** cancer cachexia, radiographic biomarker, machine learning, artificial intelligence, uncertainty, reliability, robustness

## Abstract

**Highlights:**

**What are the main findings?**

- SMAART-AI is an uncertainty-aware CT muscle analysis pipeline that combines robust segmentation with ensemble uncertainty and triage, supporting reliable automated muscle quantification across heterogeneous cancer cohorts.
- SMAART-AI enables multimodal integration of imaging-derived muscle metrics (SMA/SMI) with clinical features, improving downstream modeling for prognostic tasks (survival) and clinical endpoints (e.g., cachexia/recurrence prediction).

**What are the implications of the main findings?**

- Uncertainty-based filtering creates a transparent deployment pathway by flagging higher-risk (noisy/out-of-distribution) cases for expert review while allowing scalable automated processing for routine cases.
- CT-derived muscle biomarkers can be operationalized at scale for cachexia assessment across cancers, strengthening prognostic stratification when combined with clinical data and supporting reproducible, longitudinal monitoring.

**Abstract:**

Loss of skeletal muscle mass in cancer cachexia is associated with poorer survival, reduced treatment tolerance, and diminished quality of life. Routine oncology computed tomography (CT) can yield skeletal muscle area (SMA) and skeletal muscle index (SMI) for early cachexia assessment and prognostication, but manual annotation is labor intensive and existing automated tools often show inconsistent reliability. We developed SMAART-AI (Skeletal Muscle Assessment—Automated and Reliable Tool based on AI), a fully automated pipeline that localizes the third lumbar (L3) vertebral level, segments skeletal muscle, and quantifies prediction uncertainty to flag potentially unreliable outputs. Performance and reliability were evaluated across gastroesophageal, pancreatic, colorectal, and ovarian cancer cohorts, benchmarking against expert annotations and existing tools. SMAART-AI achieved a Dice score of 97.80% ± 0.93% in gastroesophageal cancer and a median SMA deviation of 2.48% from expert annotations across pancreatic, colorectal, and ovarian cohorts. Uncertainty scores correlated strongly with prediction error, enabling identification of high-error cases to support trustworthy deployment. Integrating the SMA/SMI with clinical features and body mass index (BMI) improved survival prediction (concordance index was +2.19% for colorectal, +9.82% for pancreatic, and +2.58% for ovarian cancer) and supported cachexia detection (70.00% accuracy; F1 80.00%). Overall, SMAART-AI provides an uncertainty-aware, clinically translatable framework for scalable CT-based muscle assessment and improved oncologic prognostication.

## 1. Introduction

Cancer cachexia, a multifactorial syndrome characterized by involuntary weight loss, skeletal muscle atrophy, and fatigue, presents a significant challenge in cancer management and represents a critical unmet need for predictive, scalable assessment methods [1]. Affecting approximately 80% of cancer patients and contributing to 20–30% of cancer-related deaths, cachexia profoundly reduces quality of life and complicates treatment [2,3,4]. Cancer cachexia is especially prevalent in gastroesophageal, pancreatic, colorectal, lung, and hematological cancers [2,5,6]. Since cancer cachexia becomes irreversible at later stages, reliable, automated estimation of skeletal muscle mass to enable early detection of the syndrome is essential to guide timely interventions that preserve muscle mass, improve treatment tolerance, and enhance survival rates [3,7,8,9,10]. This motivates the development of data-driven imaging and multimodal solutions tailored for clinical translation.

Current assessments of cachexia often utilize anthropometric measurements, such as weight, body mass index (BMI), waist circumference, and bioelectrical impedance analysis (BIA), because they are easily collected in clinical or research studies [2,3,11]. However, these measures have limitations and are insufficient for longitudinal monitoring or precision oncology. A patient’s weight might remain relatively stable despite skeletal muscle loss, and BIA is influenced by hydration status and exercise [3,11]. Computed tomography (CT), already routine in oncology, offers a more precise alternative, with skeletal muscle area (SMA) and the derived body size-adjusted metric, the skeletal muscle index (SMI), from single thoracic or abdominal slices, providing a reliable estimate of overall muscle mass [12,13,14]. Despite their efficacy, the manual extraction and annotation of these slices is laborious and irreproducible at scale. The existing automated tools are limited by inconsistent accuracy and a lack of safeguards against failure, such as uncertainty quantification or methods to detect out-of-distribution cases, which restrict their use in clinical workflows.

Conventional machine learning methods have been used to automate skeletal muscle segmentation in CT scans using various atlas-based techniques [15,16,17,18]. However, these methods depend on handcrafted features, requiring significant domain expertise and manual input, and are not robust to diverse clinical imaging protocols. Deep learning (DL) approaches, especially convolutional neural networks (CNNs), have yielded superior performance in body composition analysis by learning features directly from imaging data, enabling broader benchmarking across cancer cohorts [19]. This advancement has streamlined tissue segmentation into a two-step process: identifying the mid slice at the third lumbar level (L3) and then segmenting skeletal muscle [20,21,22,23,24,25,26,27]. However, most current models optimize for segmentation accuracy alone, without addressing reliability, uncertainty quantification, or reproducibility, features that are critical for real-world clinical deployment.

Despite their promise, existing DL models face several challenges when considered for real-world clinical use, where robustness and transparency are required alongside accuracy [28,29]. Many models trained on large datasets achieve strong performance on benchmark datasets, but often degrade in real-world settings, which may be out-of-distribution or noisy [30]. Such performance drops often arise from out-of-distribution data (e.g., scans from different populations or protocols) or noisy inputs (e.g., metal artifacts, poor image quality, or motion artifacts). Owing to their design, these DL models can fail without issuing any warning to the users, undermining clinician trust and safe adoption [31,32]. The lack of availability of model development source codes and pre-trained weights severely hampers study reproducibility. This makes it difficult for researchers to replicate results or build on prior work, ultimately limiting further research and clinical adoption [33].

Several open-source and proprietary segmentation tools are available for skeletal muscle segmentation, including SliceOmatic (version 6.0, TomoVision, Magog, QC, Canada) [34], ABACS (Automatic Body Composition Analyzer using Computed Tomography Image Segmentation available within SliceOmatic 5.0,Voronoi Health Analytics Inc., Vancouver, BC, Canada) [35], DAFS (Data Analysis Facilitation Suite, version 3.11.4, Voronoi Health Analytics Inc., Vancouver, BC, Canada) [36], AW Server (Advanced Workstation Server, version 3.2, General Electric (GE) HealthCare, Chicago, IL, United States) [37], and TotalSegmentator [38]. Among these, SliceOmatic and AW Server offer manual segmentation guided by Hounsfield windowing, while ABACS (as a plug-in used in conjunction with SliceOmatic), DAFS, and TotalSegmentator provide automated AI-based segmentation options. Although these automated tools have demonstrated an advantage over manual segmentation in terms of the time taken for the task [24,26,27], they still lack the full automation needed for clinical integration. In particular, accuracy often degrades on noisy or out-of-distribution scans without warning, and, in some cases, segmented masks cannot be retrieved for quality control or correction, limiting transparency and clinician trust.

**Figure 1:**
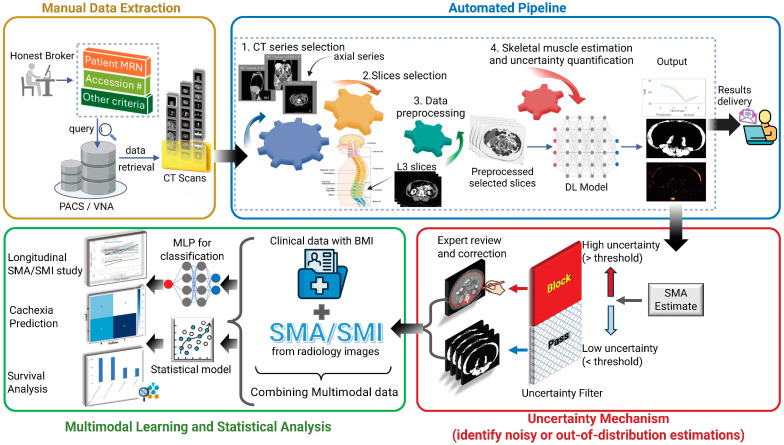
Overview of the proposed framework for Skeletal Muscle Assessment—Automated and Reliable Tool based on AI (SMAART-AI). This tool can easily be integrated into clinical workflows to assess and monitor skeletal muscle area (SMA) changes as a biomarker for cancer cachexia diagnosis. Data extraction is a manual process presented in Manual Data Extraction, where patient computed tomography (CT) scans are retrieved from the Picture Archiving and Communication System (PACS)/Vendor Neutral Archive (VNA) systems based on specific criteria. This data is then processed through the automated pipeline of SMAART-AI, starting with CT series selection (axial series) and slice selection at the third lumbar (L3) level. The selected slices undergo data preprocessing to prepare the images for being passed on to the trained deep learning (DL) segmentation model for inference. The DL model segments the skeletal muscle to estimate the SMA, generates the corresponding uncertainty map, and calculates the uncertainty metrics. An uncertainty filtering mechanism then applies thresholding to identify high- and low-uncertainty cases, blocking noisy and out-of-distribution images with a high probability of degraded performance by the DL segmentation model. The blocked images and corresponding segmentations are passed on for expert review and correction, ensuring reliable SMA and skeletal muscle index (SMI) estimations. These estimations are combined with clinical data (for example, age, height, gender, weight, body mass index (BMI), race, ethnicity, and cancer stage) to form a multimodal dataset, which is then used for multimodal learning and statistical analysis. SMA and SMI are monitored longitudinally for patients identified to be both cachectic and non-cachectic at the time of cancer diagnosis. A multilayer perceptron (MLP) model is trained for cachexia and recurrence classification, and the survival analysis shows better performance with multimodal data compared to unimodal clinical data alone.

We propose SMAART-AI (Skeletal Muscle Assessment—Automated and Reliable Tool based on AI), an end-to-end, open-source image processing and machine learning pipeline designed to address existing limitations in skeletal muscle assessment and to translate radiology-derived muscle metrics into clinically meaningful applications. Unlike prior approaches, SMAART-AI integrates ensemble-based uncertainty estimation with an uncertainty-aware triage method to ensure reliable segmentation and demonstrates robustness across heterogeneous cancer cohorts [39,40,41]. Beyond segmentation, SMAART-AI leverages accurate and reliable skeletal muscle metrics (SMA and SMI) in combination with clinical data for downstream tasks, including cachexia prediction, recurrence prediction, and survival analysis, thereby demonstrating real-world clinical utility. This comprehensive framework offers a robust solution for enhancing cancer cachexia assessment and predictive modeling by integrating radiographic biomarkers [42,43]. The schematic layout of the proposed tool and study design is depicted in Figure 1. A list of cancer cachexia biomarkers used in the literature is given in the Supplementary Materials (Table S2).

This study makes several key contributions:

1. End-to-end automated skeletal muscle quantification: Enabling reproducible and scalable assessment of cancer cachexia across cohorts and supports longitudinal patient monitoring.
2. Robust and uncertainty-aware segmentation: SMAART-AI employs a structurally diverse ensemble of nnU-Net models with random initialization to enhance robustness, particularly on noisy or out-of-distribution scans. We integrate multiple uncertainty estimation strategies and demonstrate a strong correlation between uncertainty and error, enabling performance-aware triage and reliable deployment.
3. Benchmarking against existing tools: We systematically compare SMAART-AI to widely used commercial and open-source tools (ABACS, DAFS, AW Server, and TotalSegmentator). SMAART-AI demonstrates competitive or superior accuracy while providing reproducibility safeguards, open availability, and uncertainty quantification, which are absent from proprietary solutions.
4. Clinical translation via multimodal prognostic modeling: By integrating SMA/SMI with clinical features, SMAART-AI improves prediction of cachexia, recurrence, and survival across multiple cancer types. This highlights the framework’s clinical utility as a data science-driven approach to prognostic modeling in oncology.

## 2. Materials and Methods

### 2.1. Datasets

This study utilized patient data and CT images acquired from cancer patients treated at the H. Lee Moffitt Cancer Center and Research Institute (Tampa, FL, USA). Patients from four types of cancer were included in this study, gastroesophageal cancer, colorectal cancer, pancreatic cancer and cysts, and ovarian cancer, as summarized in Table 1.

For each cancer type, the patient cohort was divided into two parts: one for model development/training and the other for evaluation as a held-out testing set. These cohorts supported three core tasks: skeletal muscle segmentation, survival analysis, and prediction of cachexia and recurrence. The segmentation task utilized all available patients per cancer type, while the survival and prediction tasks used subsets of patients with relevant clinical outcomes. Table 1 outlines the number of patients, CT scans per patient, annotated image slices (one per CT scan) used for training and testing the models, and the breakdown of the training and testing sets by cancer type for each task (segmentation, survival analysis, and prediction of cachexia and recurrence).

All CT images were stored in DICOM format. Details about DICOM processing in our framework are given in the Supplementary Materials. The pancreatic cancer cohort also included patients from the Florida Pancreas Collaborative study [40]. The Institutional Review Board reviewed and approved this research to ensure compliance with ethical standards.

#### 2.1.1. Gastroesophageal

The gastroesophageal dataset included 24 patients, each with multiple scans, one at each time point, totaling 70 scans (Table 1). Specifically, 5 patients had 2 scans, 16 had 3 scans, 2 had 4 scans, and 1 patient had 5 scans. Only the mid-L3 slices, one per scan, from the non-contrast axial series, along with the corresponding skeletal muscle masks and SMA, were used.

#### 2.1.2. Colorectal

The colorectal dataset included CT scans from 60 patients, each with one scan taken at diagnosis or before treatment/surgery. The dataset included multi-slice and multi-series CT scans with all or some combinations of views (axial, sagittal, and coronal), maximum-intensity projections, contrast, and non-contrast, and within contrast, arterial, venous, and delayed phases. Each view may have more than one series based on the available phases (such as contrast and non-contrast). The mid-L3 slices were used, one per scan. For some patients, the mid-L3 slice from more than one axial series was taken, making a total of 90 images from 60 patient scans. All 60 patients were included for survival analysis based on the availability of clinical data (Table 1) and the patient characteristics are summarized in Table 2.

#### 2.1.3. Pancreatic

The pancreatic dataset comprised 153 patients diagnosed with either malignant or benign pancreatic masses [44,45], including pre-malignant lesions, intraductal papillary mucinous neoplasms (IPMNs), pancreatic ductal adenocarcinoma (PDAC), and pancreatic neuroendocrine tumors (PNETs). A subset of patients with baseline CT scans conducted at the time of cancer diagnosis also had follow-up scans at approximately six-month intervals, and a further subset of those with first follow-up scans had scans at the second follow-up. Each time point included one CT scan per patient, resulting in 222 scans from 153 unique patients. All CT scans were multi-slice and multi-series. Axial CT series in the post-contrast venous phase, which is commonly present, was used for processing when available. For survival analysis and cachexia prediction, a subset of 130 patients with complete clinical data at diagnosis was selected, of which 89 were PDAC cases. The characteristics of these 130 patients are summarized in Table 2.

#### 2.1.4. Ovarian

The ovarian cancer cohort consisted of 324 CT scans, with each patient having one scan around the time of cancer diagnosis. A subset of 175 patients, with characteristics summarized in Table 2, was randomly selected for survival analysis and recurrence prediction due to the time-consuming nature of manual annotation of skeletal muscles (required for validation). The dataset included multi-slice and multi-series CT scans with all or some combinations of views (axial, sagittal, and coronal), maximum-intensity projections, contrast, and non-contrast, and within contrast, arterial, venous, and delayed phases. Axial CT series in the post-contrast venous phase, which is commonly present, was used for processing when available.

### 2.2. Data Processing for Ground Truth Development

In this manuscript, SliceOmatic refers to the manual tool, whereas ABACS refers to the automatic tool available as a plug-in within SliceOmatic.

#### 2.2.1. Annotations for Segmentation Model Training, Evaluation, and Comparative Analysis

Gastroesophageal cancer dataset: The SliceOmatic tool was used to create segmentation masks and calculate the SMA for the mid-L3 slice. Experts manually generated these masks using the Hounsfield unit (HU) window and made corrections using the ‘region growing’ mode for all 70 images (Table 1).

Colorectal cancer dataset: The SMA for the mid-L3 slices was estimated using DAFS, an AI-based tool by Voronoi Health Analytics. This tool automatically selected the mid-L3 slice using a proprietary algorithm. Only the SMA values were available, without the skeletal muscle mask or information about the specific mid-L3 slice images identified by DAFS. For comparison, the SMA was estimated at the manually determined mid-L3 level for 53 randomly selected CT images (Table 1) using SliceOmatic. SMAART-AI estimated the SMA for all 60 patient scans at the same manually identified mid-L3 level and at the automatically identified mid-L3 slice using the internal method explained in the Supplementary Material.

Pancreatic dataset: A radiologist calculated the SMA for the manually selected end of the L3 or start of the L4 slice (referred to as end-L3 in this study) from the axial series using the AW Server tool. Estimation of the SMA using the AW Server was based on HU windowing for muscle, and no manual correction was made. Only the SMA and end-L3 CT slice information were available, but not the pixel-level skeletal muscle mask. For comparison, the SMA for the same end-L3 slices was estimated using SliceOmatic and SMAART-AI. SMAART-AI was used for all available scans, whereas SliceOmatic was used for 109 randomly selected scans only (Table 1).

Ovarian cancer dataset: SMA values for the mid-L3 slices identified manually were evaluated using ABACS by a radiologist. ABACS, an AI-based tool, is a plug-in available within SliceOmatic (TomoVision) by Voronoi Health Analytics. The ABACS tool automatically generates skeletal muscle masks for manually selected slices. For this dataset, while SMA values and mid-L3 CT slice information were available, the skeletal muscle mask itself was not. Manual estimation of the SMA using SliceOmatic was performed for 154 randomly selected patient scans (Table 1) at the same mid-L3 slice for comparison with ABACS. SMAART-AI estimated the SMA for all available scans at the same manually identified mid-L3 level and at the automatically identified mid-L3 slice for comparison with ABACS and SliceOmatic.

#### 2.2.2. Ground Truth Development for Cancer Cachexia Detection

For the pancreatic dataset, the patients’ cachexia status was determined based on the two-stage system defined by Fearon et al. [46]. This two-stage system categorizes patients as either cachectic or non-cachectic. Cachexia was diagnosed if there was >5% weight loss over the past six months when a participant had ≥20 BMI, or >2% weight loss for patients with a BMI < 20. A clinical team determined the cachexia status independently based on medical records and patient assessments.

### 2.3. SMAART-AI Framework for Reliable Skeletal Muscle Segmentation and Metric Extraction

The SMAART-AI data processing pipeline begins by identifying the axial series in CT scans and locating slices corresponding to the third lumbar vertebral level (L3). These L3 slices are converted to PNG format and processed by the DL segmentation model, which identifies skeletal muscle pixels in each slice. The DL segmentation model then generates the pixel-level skeletal muscle segmentations along with uncertainty maps as its output. In the case of multiple scans at different time points for the same patient, a plot for longitudinal monitoring of the SMA/SMI is included in the output [47]. Additionally, a file is generated containing patient deidentified IDs, scan dates, CT series numbers, slice numbers, the corresponding SMA, quantified uncertainty values, and study/series descriptions to distinguish contrast and venous-phase axial series. A user-defined threshold on uncertainty was used to segregate expected high-error SMA predictions by the DL segmentation model in the case of out-of-distribution or noisy images. CT images with high SMA errors were manually annotated using SliceOmatic. The SMA values estimated by the DL segmentation model, along with the manually generated SMAs for high-uncertainty cases, were used alongside clinical data for downstream tasks of survival analysis, cachexia, and recurrence prediction.

#### 2.3.1. Automated Selection of Axial Series and Lumbar-Level Slice

SMAART-AI identified all axial series within the complete CT scan in DICOM format using patient orientation data from the DICOM header attribute ‘ImageOrientationPatient’. Each axial series could contain multiple groups, which were identified (if present). The image slices were sorted using DICOM header attributes such as study and series instance, slice thickness, spacing between slices, frame of reference, image position, CT series, and acquisition number.

L3 slices were then identified using the open-source tool TotalSegmentator [38] (available on GitHub: https://github.com/wasserth/TotalSegmentator accessed on 1 May 2024, version 2.0.4). TotalSegmentator segmented 117 anatomical structures in CT images and saved each segmented axial series in NIfTI format. Each anatomical structure was assigned an index number, with the L3 vertebra assigned index number 29. The identified axial series were processed by TotalSegmentator to produce segmented NIfTI files, and index number 29 was used to identify the L3 slices. Details about the softwares and hardware used in developing this pipeline are given in Supplementary Materials Table S5.

#### 2.3.2. nnU-Net for Segmentation

The nnU-Net framework was used to train and validate a DL segmentation model for identifying skeletal muscle [48]. The U-Net architecture was chosen for its exceptional performance in medical imaging segmentation tasks [49]. Since the training dataset consisted of single L3 slices, we used 2D nnU-Net architectures. Two model options with different architectures were available: ‘PlainConvUNet’ and ‘ResidualEncoderUNet.’ The architecture of PlainConvUNet included 7 encoder stages, each with 2 convolutional blocks, followed by 2 convolutional blocks in the bottleneck stage. The decoder also had 7 stages, each containing 2 convolutional blocks. Skip connections link the output of each encoder stage to the corresponding decoder stage. Each convolutional block in both encoder and decoder has a convolutional layer, followed by an instance normalization layer and a Leaky ReLU activation function. The ‘ResidualEncoderUNet’ consisted of 7 stages in both the encoder and decoder, connected by skip connections. The residual blocks were distributed as follows: (i) stage 1: 1 residual block, (ii) stage 2: 3 residual blocks, (iii) stage 3: 4 residual blocks, (iv) stages 4 to 7: 6 residual blocks each, and (v) bottleneck stage: 6 residual blocks. Each residual block contained two convolutional blocks, followed by two sets of convolutional layers, instance normalization layers, and a Leaky ReLU activation layer.

Two segmentation models were trained using the nnU-Net framework with 5-fold cross-validation. For each fold, the training started with randomly initialized weights and ran for 1000 epochs with a learning rate of 0.01. The training dataset consisted of 45 mid-L3 slice images from the gastroesophageal dataset and 15 end-L3 (end of L3 and before start of L4) slice images from the pancreatic cancer dataset. We used data augmentation techniques built into the nnU-Net framework, including random rotations, scaling, intensity shifts, and flipping. These augmentations are applied dynamically during training and are designed specifically for medical image segmentation tasks to improve model robustness and generalizability. The held-out test set included 25 images from the gastroesophageal dataset, and the performance of the DL segmentation model was evaluated using the average Dice score and Jaccard index, which have been reported in Section 3. Additionally, the trained segmentation model was used to run inference on the pancreatic, colorectal, and ovarian datasets (Table 1).

The pixel-wise average probability from the output of the models across the 5 folds was calculated, and all pixels with an average probability greater than 0.50 were marked as skeletal muscle. There are cases termed as false positives and false negatives. False positive pixels were not part of the skeletal muscle in the manually marked mask; however, the DL segmentation model identified the pixels as part of it. False negative pixels were part of the skeletal muscle, but the model did not mark them as part of the skeletal muscle mask it produced.

#### 2.3.3. Uncertainty Estimation Methods and Metrics

In DL, two primary sources of predictive uncertainty are commonly considered: aleatoric uncertainty, which reflects irreducible noise in the data, and epistemic uncertainty, which reflects uncertainty in model parameters and can be reduced with additional diverse training data. To estimate uncertainty in SMA estimations, we compared three approaches, post hoc calibration, Monte Carlo dropout, and deep ensembles, and computed multiple uncertainty metrics that quantify total uncertainty (aleatoric + epistemic), epistemic uncertainty, and (where applicable) aleatoric uncertainty. Ensemble-based uncertainty was used for reliability triage across cohorts. Monte Carlo dropout was evaluated only in the gastroesophageal cohort as a sensitivity analysis to confirm that the uncertainty and SMA estimation error relationship is consistent under an alternative epistemic estimator. Given the dropout approach’s slightly lower segmentation agreement (Table 3) and higher inference cost, it was not propagated to other cohorts. Post hoc calibration was included as a comparative analysis to assess whether calibrated probabilities improve alignment between model confidence metrics and SMA estimation error.

1. Post hoc Calibration: The ‘netcal’ Python library (version 1.3.6) [50] with ’LogisticCalibration’ (Platt scaling) was used. The calibration model was trained using the DL model outputs and corresponding labels. During inference, the DL model outputs were passed through the calibration model to obtain calibrated probabilities.
2. Monte Carlo Dropout: A dropout layer (*p* = 0.20) was added after each convolutional layer in the ‘ResidualEncoderUNet’ architecture. The model was trained with 5-fold cross-validation, and inference was repeated 20 times per fold. At each iteration, the average of the 5-fold ensemble predictions was calculated. The final dropout prediction was the pixel-wise mean of these stochastic predictions, and uncertainty was computed as the mean pixel-wise variance across them.
3. Model Ensemble: Ten models were used, five ‘PlainConvUNet’ and five ’ResidualEncoderUNet’, corresponding to 5-fold cross-validation for each architecture. The final prediction was the pixel-wise mean across the ten models, and uncertainty was computed from the mean pixel-wise variance across model outputs.

The following metrics were used for quantifying the uncertainty estimated using the different techniques [51]:

1. Average Probability: Calculated by taking the average of the output probabilities of the predicted class at each pixel in a single image. This metric captures the total uncertainty.
2. Average probability (SM): This is the average output probability of pixels marked as skeletal muscle (SM) only. This metric captures the total uncertainty.
3. Average Calibrated Probability: Average of the calibrated output probabilities of the predicted class at each pixel in a single image. This metric captures the total uncertainty.
4. Coefficient of Variation (pixel-wise): The average of the pixel-wise coefficient of variation, calculated from the ensemble or dropout outputs as the ratio of the standard deviation (SD) to the mean. The average pixel-wise coefficient of variation was computed as the ratio of the standard deviation to the mean of the predicted probability across ensemble models or dropout passes. This metric captures the epistemic uncertainty.
5. Coefficient of Variation (SMA): Calculated using the standard deviation and mean of the SMA estimated by each model in the ensemble or multiple inferences in case of the dropout method. This metric captures the epistemic uncertainty.
6. Average Variance: It is calculated as the average of the variance computed for each pixel. The pixel-wise variance is calculated using the output probabilities from the ensemble models or multiple inferences using the dropout method. This metric captures the epistemic uncertainty.
7. Average Variance (SM): Average of the variance for pixels identified as being part of the skeletal muscle (SM) only. This metric captures the epistemic uncertainty.
8. Average Entropy: Estimates the total uncertainty by calculating the binary entropy at each pixel based on the average output probabilities across pixels in either an ensemble of models or multiple inferences with dropout. The average entropy of all pixels across the image is reported.
9. Expected Entropy of the Ensemble: Estimates aleatoric uncertainty by calculating the binary entropy at each pixel for all the models in the ensemble. The average entropy is computed for each pixel across all models, and the final reported value is the mean of these pixel-wise average entropies across the entire image.

#### 2.3.4. Statistical Tests for Uncertainty Methods and Metrics

The Pearson correlation coefficient (r) was calculated between each uncertainty method/metric and the difference between the SMA estimated by SMAART-AI and that estimated by SliceOmatic. The interpretation of r values is as follows: |r| = 0 indicates no relationship, 0 < |r| ≤ 0.3 indicates a weak relationship, 0.3 < |r| ≤ 0.5 indicates a moderate relationship, 0.5 < |r| ≤ 0.7 indicates a strong relationship, |r| > 0.7 indicates a very strong relationship, and |r| = 1 represents a perfect relationship. The statistical significance of these correlations was assessed using Student’s *t*-test, with a significance level set at 95% (*p* < 0.05 indicating statistical significance). This analysis established the degree of association between each uncertainty method/metric and the error in SMA estimated by SMAART-AI, providing insight into how well each method can identify cases with potentially high estimation errors.

#### 2.3.5. Methods for Identifying High-Error SMA Predictions in SMAART-AI

The ensemble approach, together with the average variance and coefficient of variation (SMA) uncertainty metrics, was used to identify cases with a higher likelihood of SMA estimation error across the colorectal, pancreatic, and ovarian cohorts. A dataset-specific uncertainty threshold was applied to flag cases for review, capturing both underestimation and overestimation of SMA. For evaluation, high-error cases were defined as those with >2.5% SMA difference relative to SliceOmatic.

Uncertainty thresholds were defined per cohort to support practical triage using one of two criteria: (i) quantile-based thresholding, where the threshold is set to a chosen percentile of the cohort’s uncertainty distribution to control the proportion of CT images referred for review; and/or (ii) validation-based thresholding, where a held-out split (using SliceOmatic as reference) is labeled as high-error vs. low-error and candidate thresholds are evaluated to balance the detection of high-error cases against review burden. Lower thresholds increase the detection of high-error cases but increase false positives, whereas higher thresholds reduce false positives but miss more high-error cases. We report the percentile rank of each threshold and the fraction of images flagged.

### 2.4. Survival Analysis Using SMAART-AI

For survival analysis, we used the ‘CoxPHFitter’ tool from the ‘lifelines’ Python library (version 0.30.1) on three datasets: pancreatic, colorectal, and ovarian cancer [52]. The data included clinical variables such as age, gender, race, ethnicity, weight, height, cancer stage, BMI, CT-derived metrics including SMI and SMA, time to event (TTE), and vital status. was Analyses were performed using unimodal clinical variables available around the time of cancer diagnosis and multimodal data combinations integrating clinical variables with radiology derived SMA and SMI features [53,54,55,56,57,58]. Multiple penalizer values were evaluated in ‘CoxPHFitter’ tool to identify the optimal model for each combination of SMA, SMI, and BMI, and the best-performing results are reported. The number of patients in the training and held-out test sets are given in Table 1. Confidence intervals were estimated by patient-level bootstrap resampling of the held-out test set using 2000 iterations, with 95% confidence intervals derived from the empirical bootstrap distribution.

### 2.5. Cancer Cachexia Prediction Using SMAART-AI

Cachexia prediction was formulated as a binary classification task for the pancreatic dataset. We trained a multilayer perceptron (MLP) with three fully connected layers (256, 128, and 32 nodes), each followed by ReLU activation and dropout, and a final sigmoid output layer. Dropout probabilities were 0.20 after the first two layers and 0.50 after the third layer. The model was trained for 50 epochs with a learning rate of 5 × 10^−5^ using 5-fold cross-validation, and final predictions were obtained by averaging across folds. Of the 130 pancreatic patients used for prediction model development, 100 were allocated to training and validation (85:15 split), while an independent held-out test set of 30 PDAC patients (Table 1) was used for evaluation. In this cohort, 70 patients were classified as cachectic and 60 as non-cachectic. Confidence intervals (CIs) were estimated by patient-level bootstrap resampling of the held-out test set using 2000 iterations. Point estimates were reported on the original held-out test set, and 95% confidence intervals were derived from the empirical bootstrap distribution.

### 2.6. Recurrence Prediction Using SMAART-AI

Recurrence prediction was formulated as a binary classification task for the ovarian dataset. We trained a multilayer perceptron (MLP) with three fully connected layers (64, 32, and 16 nodes), each followed by ReLU activation and dropout, and a final sigmoid output layer. Dropout probabilities were 0.75, 0.50, and 0.65 after the first, second, and third layers, respectively. The model was trained for 200 epochs with a learning rate of 5 × 10^−4^ using 5-fold cross-validation, and final predictions were obtained by averaging across folds. The dataset consisted of 175 ovarian cancer patients, split into 125 for training/validation (85:15 ratio) and 50 as a held-out test set for model evaluation (Table 1). To address class imbalance in the training set, we applied the Synthetic Minority Over-sampling Technique (SMOTE) [59]. In this cohort, 116 patients experienced recurrence, whereas 59 did not. Confidence intervals (CIs) were estimated by patient-level bootstrap resampling of the held-out test set using 2000 iterations. Point estimates are reported on the original held-out test set, and 95% confidence intervals were derived from the empirical bootstrap distribution.

## 3. Results

### 3.1. Comparison of the Predicted SMA Between SMAART-AI, TotalSegmentator, DAFS, ABACS, AW Server, and SliceOmatic

#### 3.1.1. Gastroesophageal Dataset (Comparison of SMAART-AI and the Ground Truth Masks Generated by Experts Using SliceOmatic)

Table 3 compares the SMA estimated by SMAART-AI, using both ensemble and dropout techniques, with the ground truth SMA estimated manually by experts using SliceOmatic. The SMA is reported as the pixel count classified as skeletal muscle, serving as a proxy for SMA measurement. With the ensemble technique, the mean and median absolute differences between SMAART-AI and SliceOmatic were 2.44% and 0.81%, respectively. The corresponding mean and median Jaccard indices were 94.21% and 94.84%, while the Dice scores were 96.96% and 97.35%. Using the dropout technique, the mean and median absolute differences were 2.72% and 1.06%, with mean and median Jaccard indices of 93.95% and 94.93%, and Dice scores of 96.82% and 97.40%. The false positive count indicates the number of pixels marked as skeletal muscle by SMAART-AI but not included in the skeletal muscle mask generated manually using SliceOmatic. Conversely, the false negative count represents pixels missed by SMAART-AI that were included in the skeletal muscle mask generated manually using SliceOmatic. Cases highlighted in red correspond to noisy or out-of-distribution CT images, which resulted in degraded performance by SMAART-AI.

For both the dropout and ensemble techniques, the average Jaccard index, excluding the four out-of-distribution images highlighted in red, was 95.52 and 95.71, respectively, and the average Dice score was 97.70 and 97.80. The average difference in estimated SMA was 1.02% for the dropout method and 0.90% for the ensemble method. Overall, the ensemble method performed better than the dropout method.

**Table 3:** Performance Comparison of SMAART-AI (ensemble and dropout techniques) and the ground truth generated by manual segmentation using SliceOmatic for skeletal muscle area (SMA) estimation for the gastroesophageal cancer test dataset.

	SMAART-AI—Ensemble of Models					SMAART-AI—Dropout Technique				
Patient ID.scan	Area Difference Model vs. SliceOmatic (%)	Jaccard Score (%)	Dice Score (%)	False Positive	False Negative	Area Difference Model vs. SliceOmatic (%)	Jaccard Score (%)	Dice Score (%)	False Positive	False Negative
2.2	0.89	97.39	98.68	381	189	0.586	97.64	98.81	320	194
2.4	−0.16	96.98	98.47	262	291	2.360	95.44	97.67	639	213
3.1	0.79	94.14	96.98	701	539	−1.056	91.45	95.54	800	1016
4.1	0.17	98.98	99.49	119	86	0.240	98.96	99.48	129	81
4.2	1.67	97.04	98.50	505	147	2.149	96.86	98.41	577	115
5.1	−1.13	94.52	97.18	298	448	−1.277	94.61	97.23	281	451
5.2	−0.76	95.10	97.49	272	369	−0.820	95.72	97.82	226	331
5.3	−0.75	94.54	97.19	374	489	−0.052	94.21	97.02	456	464
5.4	−0.37	94.59	97.22	429	490	0.272	93.08	96.42	617	572
7.3	5.17	86.69	92.87	2916	1393	6.251	86.56	92.80	3108	1267
9.1	−0.66	97.28	98.62	197	323	−1.451	97.41	98.69	109	384
9.2	1.95	95.01	97.44	669	302	2.288	94.93	97.40	709	279
9.3	11.14	86.11	92.53	3041	523	12.427	84.90	91.83	3366	556
15.1	5.87	92.58	96.14	488	73	5.504	93.33	96.55	445	56
15.2	0.83	98.26	99.12	259	93	0.496	98.33	99.16	218	119
15.3	−1.97	96.90	98.42	106	471	−1.881	96.73	98.34	131	479
15.4	2.64	92.34	96.02	1100	557	2.757	92.86	96.30	1054	488
15.5	−0.81	92.98	96.36	701	877	0.597	93.36	96.56	815	685
16.1	−0.18	97.94	98.96	172	205	−0.320	97.85	98.91	168	226
16.2	1.54	94.12	96.97	729	436	1.799	93.88	96.84	779	436
16.3	−0.80	96.21	98.07	238	363	−0.436	96.22	98.07	266	334
21.1	0.52	96.85	98.40	125	90	0.328	97.27	98.62	104	82
21.3	−0.24	94.84	97.35	481	527	−0.173	94.93	97.40	479	512
21.5	−0.10	93.99	96.90	612	612	0.015	94.21	97.02	582	579
23.2	19.85	79.75	88.73	4170	460	22.56	78.11	87.71	4665	448

The rows in red represent noisy or out-of-distribution images.

#### 3.1.2. Colorectal Dataset (Comparison of SMAART-AI, SliceOmatic, DAFS, and TotalSegmentator)

**Figure 2:**
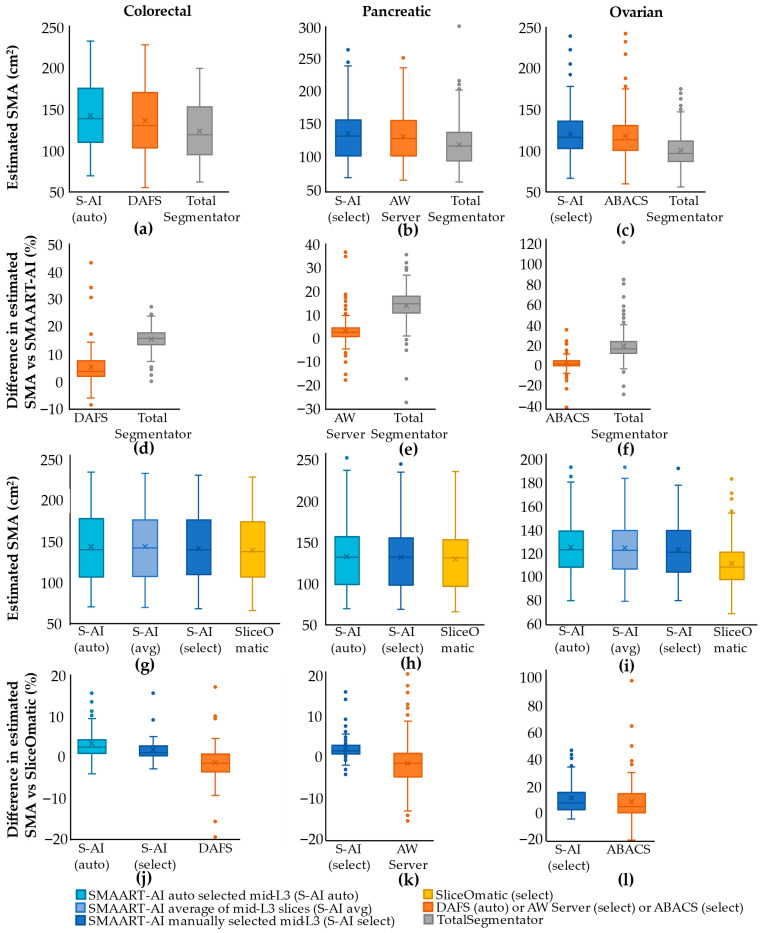
Comparative analysis and benchmarking of SMA estimation tools across datasets. The columns present results from different cancers: colorectal, pancreatic, and ovarian. The rows represent various estimates of SMA. The first two rows (subfigures (**a**–**f**)) compare the SMA estimates from different tools, and the third and fourth rows (subfigures (**g**–**l**)) benchmark SMAART-AI, DAFS, AW Server, and ABACS against SliceOmatic. Row 1: Box plots show SMA distributions estimated using various tools. Row 2: Box plots display differences between each tool and SMAART-AI. The SMA is estimated at the manually selected mid-L3 slice for AW Server and ABACS, but at the automatically selected mid-L3 slice by DAFS. Median values are close to zero for DAFS, AW Server, and ABACS, though DAFS has a few large positive outliers, whereas AW Server and ABACS show balanced over- and underestimation. The median for TotalSegmentator is relatively high, showing underestimation. Row 3: Differences in SMA estimates from SliceOmatic and SMAART-AI (using three methods for mid-L3 slice selection). These methods include: (1) automatically selected mid-L3 slice (S-AI (auto)), (2) average across adjacent slices (S-AI (avg)), and (3) manually selected mid-L3 slice (S-AI (select)). Estimates from S-AI (auto) and S-AI (avg) are nearly identical, both slightly higher than S-AI (select) across the datasets. SMAART-AI closely matches SliceOmatic at the manually identified mid-L3 slice in colorectal and pancreatic datasets, but overestimates in ovarian cases due to noisy images. Row 4: Distributions of differences comparing SliceOmatic with other methods. SMAART-AI has median values close to zero for colorectal and pancreatic datasets, but higher for ovarian. Compared with SliceOmatic, DAFS and AW Server slightly underestimate, ABACS overestimates, while SMAART-AI shows a smaller spread than all three, indicating greater stability. Overall, SMAART-AI performs on par or better than the other tested tools, including DAFS, AW Server, and ABACS, when benchmarked using SliceOmatic.

The first column in Figure 2a,d,g,j presents the results for the colorectal cancer test dataset. Figure 2a shows the distribution of the SMA estimated, using different tools, on the mid-L3 slice images. DAFS and SMAART-AI determine the mid-slice using their respective automated methods, while TotalSegmentator uses the mid-slice determined by SMAART-AI. The mean/median SMA estimated using DAFS is 137.16 cm^2^/131.28 cm^2^, 124.11 cm^2^/120.16 cm^2^ using TotalSegmentator, and 143.49 cm^2^/139.37 cm^2^ using SMAART-AI. The box plots in Figure 2d compare the difference in SMA distribution for the 90 mid-L3 slices. The mean and median of the absolute differences in SMA between DAFS and SMAART-AI are 6.02% and 4.07%, respectively, while for TotalSegmentator and SMAART-AI, these are 15.58% and 15.73%, respectively.

Figure 2g presents the SMA estimates from SliceOmatic (using one image per patient scan at the mid-L3 level) and SMAART-AI (using three different approaches). These approaches include: (1) automatically determined mid-L3 by SMAART-AI (S-AI(auto)), (2) average area of slices adjacent to the mid-L3 determined by SMAART-AI (S-AI(avg)), and (3) manually selected mid-L3 (S-AI (select)). When comparing the individual SMA estimates across the three approaches using SMAART-AI, there is little difference between the mid-L3 SMA and the average SMA around mid-L3, with the average SMA having a mean/median of 143.22 cm^2^/141.81 cm^2^, which closely matches SMAART-AI’s estimate for the automatically determined mid-slice 143.42 cm^2^/139.82 cm^2^. The mean/median SMAs at the manually selected mid-L3 are 138.71 cm^2^/137.40 cm^2^ using SliceOmatic and 141.01 cm^2^/139.75 cm^2^ using SMAART-AI.

**Figure 3:**
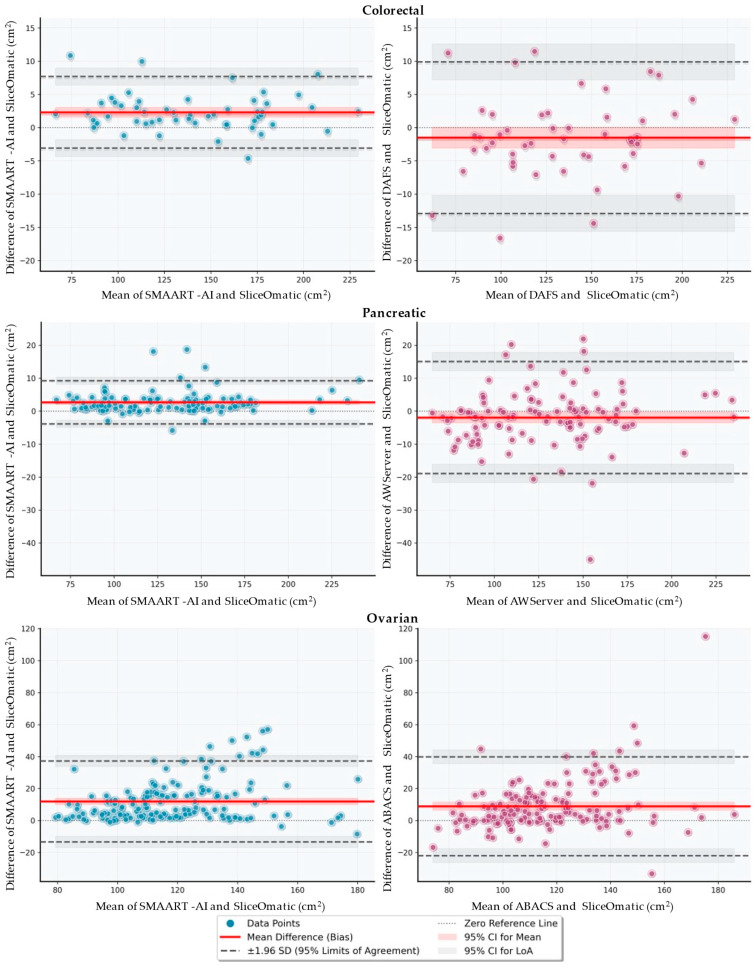
Bland–Altman plots comparing SMA estimates from SMAART-AI and commercial tools against SliceOmatic (manual reference) in colorectal (top row), pancreatic (middle row), and ovarian (bottom row) cohorts. The *x*-axis shows the mean of the paired measurements, and the *y*-axis shows the difference (comparator—SliceOmatic, cm^2^). Solid red lines denote mean bias; dashed gray lines denote 95% limits of agreement (bias ± 1.96 SD). Across cohorts, the Bland–Altman plots indicate that SMAART-AI tends to trade modest systematic bias for improved consistency, yielding tighter agreement with the manual reference than DAFS and AW Server in colorectal and pancreatic datasets. In contrast, ovarian cases showed larger spread and overestimation for both SMAART-AI and ABACS, highlighting the challenge of noisy/out-of-distribution scans and the importance of uncertainty-aware review.

Figure 2j presents distributions of the absolute difference in SMA between the ground truth (SliceOmatic) and S-AI (auto), S-AI (select), and DAFS. The mean and median absolute difference between SMAART-AI (auto) and SliceOmatic are 3.97% and 2.73%, respectively. For SMAART-AI (select), the absolute difference between the SMA estimated by SMAART-AI and SliceOmatic has a mean of 2.21% and a median of 1.38%. The absolute difference between the estimations by DAFS versus SliceOmatic has a mean of 3.77% and a median of 2.36%. We evaluated the agreement of SMAART-AI, DAFS, and SliceOmatic using Bland–Altman analysis [60]. As presented in Figure 3, compared with SliceOmatic, SMAART-AI demonstrated a mean bias of +2.30 (95% limits of agreement (LoAs): −3.08 to +7.68), indicating slightly higher SMA estimates on average with relatively narrow limits of agreement. In contrast, DAFS showed a mean bias of −1.51 (95% LoAs: −12.92 to +9.89), suggesting closer average alignment with SliceOmatic but substantially greater variability. These results indicate that while DAFS is closer to SliceOmatic on average, SMAART-AI provides more consistent agreement with a smaller dispersion of differences, making it more reliable for downstream analyses.

SMAART-AI performed well, with less than a 2.5% difference compared to SliceOmatic in 68% (*n* = 36) estimates when the mid-L3 slice was manually selected, though this dropped to 42% (*n* = 22) when SMAART-AI automatically selected the slice. DAFS has 53% (*n* = 28) SMA estimates with less than 2.5% difference compared to SliceOmatic. Most of the considerable differences in the estimated SMA by SMAART-AI occur in CT images that are out-of-distribution or have varying levels of noise (refer to Section 3.5)). Comparative analysis of SMA estimation using different tools for colorectal cancer is given in Supplementary Figure S1, whereas benchmarking SMA estimation by SMAART-AI versus SliceOmatic for colorectal cancer is given in Supplementary Figure S2.

#### 3.1.3. Pancreatic Dataset (Comparing SMAART-AI, SliceOmatic, AW Server, and TotalSegmentator)

The second column in Figure 2b,e,h,k presents the results for the pancreatic test dataset. Figure 2b presents a comparison of the SMA estimations made by SMAART-AI, AW Server, and TotalSegmentator. The results indicate that TotalSegmentator slightly underestimates the SMA, while SMAART-AI and AW Server provide similar values. The mean and median estimated areas are 118.81 cm^2^ and 116.97 cm^2^ for TotalSegmentator, 135.02 cm^2^ and 131.64 cm^2^ for SMAART-AI, and 131.00 cm^2^ and 127.70 cm^2^ for the AW Server. Figure 2e presents box plots of the difference between the SMA estimated by SMAART-AI, AW Server and TotalSegmentator. The mean and median absolute differences between SMAART-AI and AW Server are 4.37% and 3.04%, respectively, while the absolute differences between SMAART-AI and TotalSegmentator are 14.38% and 14.82%.

Figure 2h presents SMA estimates by SliceOmatic, SMAART-AI (auto) and SMAART-AI (select). The mean and median SMAs estimated by SMAART-AI (auto, that is, using the automatically selected end-L3 slice) are 134.12 cm^2^ and 131.95 cm^2^. The mean and median estimated SMAs for SMAART-AI (select), AW Server, and SliceOmatic are 133.45 cm^2^ and 131.95 cm^2^, 130.89 cm^2^ and 133.00 cm^2^, and 129.39 cm^2^ and 131.60 cm^2^, respectively.

Figure 2k presents the distribution of the absolute differences between SliceOmatic and AW Server, and SliceOmatic and SMAART-AI. SMAART-AI and AW Server show close agreement with SliceOmatic in approximately 87% and 61% of the cases, respectively. Bland–Altman analysis, presented in Figure 3, revealed that SMAART-AI exhibited a mean bias of +2.70 (95% LoAs: –3.85 to +9.25), indicating slightly higher estimates but with narrow limits of agreement and strong consistency. In contrast, AW Server demonstrated a mean bias of –1.92 (95% LoAs: –18.91 to +15.08), reflecting closer average alignment but substantially wider variability. These findings indicate that although SMAART-AI estimates are systematically but modestly higher than manual values, its greater consistency and narrower limits of agreement make it more reliable for downstream analyses than AW Server.

SMAART-AI consistently matched manual SliceOmatic segmentation within a 2.5% SMA difference in 67% (*n* = 73) of cases when the end-L3 slice was identified manually and in 54% (*n* = 59) of cases when the end-L3 slice was identified automatically by SMAART-AI, outperforming AW Server (43%, *n* = 47). Comparative analysis of SMA estimation using different tools for the pancreatic cancer dataset is given in Supplementary Figure S3, and benchmarking SMA estimation by SMAART-AI and AW Server versus SliceOmatic for the pancreatic cancer dataset is given in Supplementary Figure S4.

#### 3.1.4. Ovarian Dataset (Comparing SMAART-AI, SliceOmatic, ABACS, and TotalSegmentator)

The third column in Figure 2c,f,i,l presents the results for the ovarian cancer test dataset. Figure 2c presents the SMA estimates from SMAART-AI, ABACS, and TotalSegmentator. The results reveal that TotalSegmentator slightly underestimates the SMA compared to ABACS and SMAART-AI. The mean and median estimated SMAs are 120.85 cm^2^ and 116.89 cm^2^ using SMAART-AI, 118.14 cm^2^ and 114.2 cm^2^ using ABACS, and 100.90 cm^2^ and 97.54 cm^2^ using TotalSegmentator. Figure 2f shows the distribution of the differences in SMA between SMAART-AI, ABACS, and TotalSegmentator. The median and mean absolute differences between SMAART-AI and ABACS are 4.96% and 3.23%, respectively, while the median and mean absolute differences between SMAART-AI and TotalSegmentator are 20.66% and 17.31%, respectively. In 34% of the SMA estimations, the absolute difference between SMAART-AI versus ABACS is less than or equal to 2.5%.

Figure 2i presents the comparison of SMA estimates using SliceOmatic and SMAART-AI (auto), SMAART-AI (avg), and SMAART-AI (select). The mean and median SMA values are 125.78 cm^2^ and 123.55 cm^2^ using SMAART-AI (auto), 125.46 cm^2^ and 123.42 cm^2^ for SMAART-AI (avg), 123.98 cm^2^ and 121.49 cm^2^ for SMAART-AI (select), 120.98 cm^2^ and 118.25 cm^2^ for ABACS, and 112.01 cm^2^ and 108.85 cm^2^ for SliceOmatic. Figure 2l presents the distribution of SMA differences between SliceOmatic, SMAART-AI (select) and ABACS. The mean and median absolute differences in SMA between ABACS and SliceOmatic are 10.32% and 6.21%, respectively, while the mean and median absolute differences between SMAART-AI and SliceOmatic are 11.09% and 7.08%. In the Bland–Altman analysis, presented in Figure 3, SMAART-AI demonstrated a mean bias of +11.97 (95% LoAs: −13.34 to +37.28), indicating systematically higher SMA estimates with moderate variability. In contrast, ABACS showed a mean bias of +8.96 (95% LoA: −21.97 to +39.89), also reflecting overestimation relative to SliceOmatic but with greater dispersion of differences. These results suggest that while both SMAART-AI and ABACS tend to overestimate the SMA compared to manual assessment, SMAART-AI provides more consistent agreement (narrower LoA), whereas ABACS exhibits smaller average bias but greater variability.

**Figure 4:**
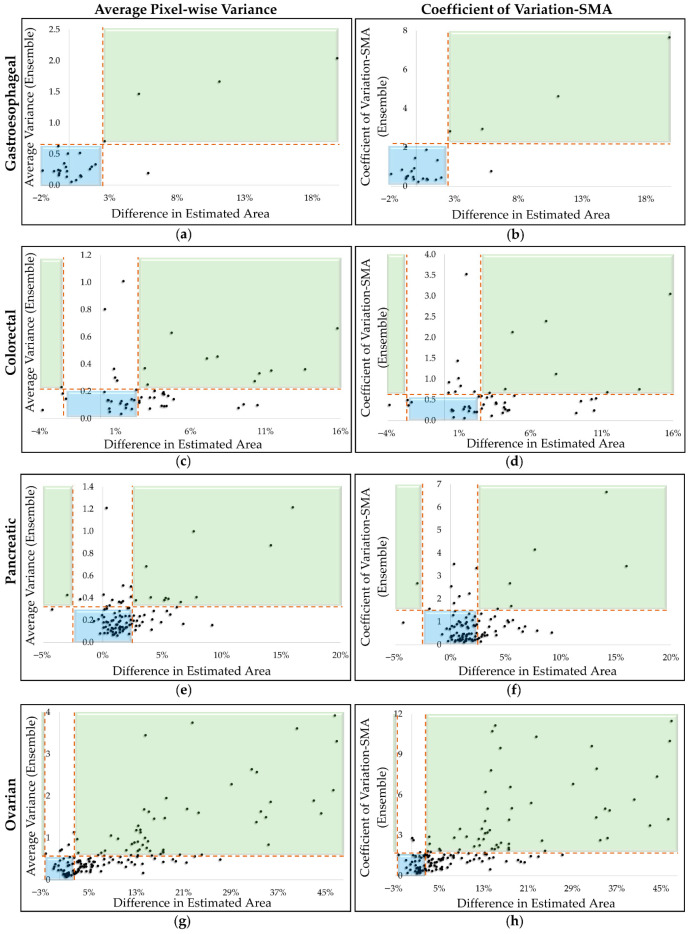
Uncertainty scatter plots for the four cancer datasets and two metrics, average variance (ensemble) and coefficient of variation (SMA). The subfigures (**a**,**c**,**e**,**g**) display the average pixel-wise variance of the estimated SMA from the ensemble, while subfigures (**b**,**d**,**f**,**h**) show the coefficient of variation in the estimated SMA using the ensemble method. The horizontal dashed line represents an adjustable uncertainty threshold, which can be used to identify cases where SMAART-AI’s estimated SMA may have high errors. The green and blue quadrants highlight required segregation: the blue quadrant represents low-difference, low-uncertainty cases, and the green quadrant represents high-difference, high-uncertainty cases. Cases in the other two quadrants fall under either low-difference, high-variance, or high-difference, low-variance categories. Both uncertainty estimation metrics exhibit different spreads of uncertain cases. Due to this difference, the efficiency of the thresholding method for identifying potentially high-error cases differs slightly within and between different datasets.

ABACS performed better in more cases overall, 32% (*n* = 50) within a difference of 2.5% from SliceOmatic, compared to 26% (*n* = 40) by SMAART-AI when the mid-L3 slice was identified manually and 18% (*n* = 28) when the mid-L3 slices were identified automatically by SMAART-AI. Nevertheless, SMAART-AI made accurate estimations in comparison to ABACS in certain images given in Figure 4c and Section 3.5. Comparative analysis of SMA estimation using different tools for ovarian cancer is given in Supplementary Figure S5, and benchmarking of SMA estimation by SMAART-AI and ABACS versus SliceOmatic for ovarian cancer is given in Supplementary Figure S6.

### 3.2. Comparison of the Uncertainty Methods and Metrics

#### 3.2.1. Correlation Between Model Uncertainty and SMA Estimation Difference

Table 4 presents the correlation coefficients between various uncertainty metrics and the difference between the SMAART-AI estimated SMA and the manually measured SMA from SliceOmatic. With the dropout method, the results show a very strong correlation (r > 0.7) for all metrics, except for the coefficient of variation (SMA), which exhibits a weak correlation (r = 0.296).

Using the ensemble method, all metrics and the calibration method demonstrate very strong correlations, with coefficients exceeding 0.7 for the gastroesophageal dataset. The colorectal cancer dataset also shows very strong correlations for the coefficient of variation (SMA) estimated from the ensemble, and strong correlations (r > 0.5) for the average variance (overall and for skeletal muscle pixels), as well as the coefficient of variation (pixel-wise). The pancreatic cancer dataset exhibits strong correlations (r > 0.5) for most metrics, except for the average calibrated probability and expected entropy of the ensemble, which show moderate correlations below 0.4. In the ovarian cancer dataset, correlations are very strong across all metrics, with values above 0.7, and strong (r > 0.6) for the coefficient of variation (SMA) and expected entropy of the ensemble.

The dropout method was tested only on the gastroesophageal dataset, as its overall performance in estimating the SMA was slightly weaker than that of the ensemble method (refer to Table 3).

#### 3.2.2. Model Uncertainty for Detecting Performance Degradation

From Table 4, we selected two representative metrics, average variance and coefficient of variation (SMA), which strongly correlated with the difference in estimated SMA. Figure 4 presents scatter plots of these metrics derived using the ensemble method to demonstrate their utility in identifying high-error cases using thresholding. High error was defined as >2.5% SMA difference relative to SliceOmatic.

In Figure 4, the green and blue shaded regions represent ideal outcomes: the green region indicates cases with high error and high uncertainty, and the blue region indicates cases with low error and low uncertainty. The horizontal dashed line represents the uncertainty threshold, and the vertical dashed line separates high- vs. low-error cases. The upper white region contains low-error cases that exceed the threshold (false positives), whereas the lower white region contains high-error cases with low uncertainty (false negatives). Table 5 summarizes the operating points (flagged fraction, threshold percentile, sensitivity, and specificity) across cohorts. The corresponding confusion matrix counts (true positive (TP)/false positive (FP)/false negative (FN)/true negative (TN)) and below/above-threshold counts for each operating point are provided in the Supplementary Materials in Table S1.

For the gastroesophageal dataset (Figure 4a,b), the coefficient of variation performed better than average variance. With the coefficient of variation, four out of five cases with an error greater than 2.5% were correctly flagged and only one low-error case was misclassified above the threshold of 2.00. Using average variance, with a threshold set to 0.50, four out of five high-error cases were correctly flagged, and three low-error cases were misclassified, showing reduced specificity compared to the coefficient of variation. For the colorectal dataset (Figure 4c,d), both metrics performed equally well. With average variance at a threshold of 0.12, 17 cases fell below the threshold and 36 above; of these, 9/17 below-threshold cases had a difference in estimated SMA greater than 2.5%, while 14/36 above-threshold cases had a difference less than 2.5%. Increasing the threshold to 0.2 led to 35 below-threshold cases (19 with a difference greater than 2.5%) and 18 above-threshold cases (six with a difference less than 2.5%). With the coefficient of variation at 0.3, 17 cases were below (eight high-difference) and 36 above (13 low-difference). At a threshold of 0.5, 30 were below (17 cases with a high difference greater than 2.5%) and 23 were above-threshold (nine with a low difference less than 2.5%).

For the pancreatic dataset (Figure 4e,f), average variance performed better than coefficient of variation. Using average variance at 0.2, 53 cases were above the threshold (30 low-difference) and 56 were below (12 high-difference). At a higher threshold of 0.4, 16 cases were above (seven low-difference) and 93 were below (27 high-difference). With the coefficient of variation at 0.6, 54 cases were above (32 low-difference) and 55 were below (13 high-difference). At a threshold of 1.0, 32 were above (17 low-difference) and 77 were below (20 high-difference).

For the ovarian dataset (Figure 4g,h), both metrics performed comparably. With the average variance threshold at 0.35, 102 cases were above (12 low-difference) and 52 were below (23 high-difference). Increasing the threshold value to 0.6 yielded 56 above (four low-difference) and 98 below (62 high-difference). Using the coefficient of variation at a threshold of 1.0, 109 cases were above (17 low-difference) and 45 were below (20 high-difference). At a higher threshold of 1.5, 71 were above (7 low-difference) and 83 were below (48 high-difference).

### 3.3. Survival Analysis

Table 6 presents the test-set concordance index (C-index) with 95% confidence intervals for the three cancer cohorts and four feature combinations. The results show that adding the SMI and SMA to clinical data improves the concordance index, with increases of 2.19%, 4.66%, and 2.58% for the colorectal, pancreatic, and ovarian datasets. In the pancreatic cohort, the highest test C-index was observed when the BMI along with the SMI and SMA were added to the clinical data. In the ovarian cohort, models including the SMI and SMA (with or without BMI) showed the highest point estimate, while the addition of BMI and clinical data only achieved an identical concordance index, indicating no measurable incremental effect of BMI in this cohort. In the colorectal cohort, the highest point estimate was obtained when the SMI and SMA were added to clinical data, while the addition of BMI reduced the concordance index by 8.76%.

Across all cohorts, the 95% confidence intervals were broad and substantially overlapping, particularly for colorectal cancer, indicating uncertainty in the magnitude of the observed differences. Consistent with this, formal statistical comparisons (paired bootstrap comparisons of test-set C-index and nested Cox likelihood ratio tests, where applicable) did not show statistically significant differences between feature combinations (all *p* > 0.05). Accordingly, these survival results should be interpreted as exploratory, with point estimate trends suggesting possible incremental value in some cohorts (especially pancreatic) that require validation in larger datasets.

### 3.4. Cancer Cachexia and Recurrence Prediction

The MLP model for cachexia prediction in pancreatic cancer patients achieved an accuracy of 70.00% (95% CI: [56.67–83.33]) on the held-out test. The model predicts whether a patient is cachectic with a precision of 72.00% (95% CI: [64.00–82.61]), recall of 90.00% (95% CI: [75.00–100.00]), and F1 score of 80.00% (95% CI: [71.11–88.39]) at the time of diagnosis.

For recurrence prediction in ovarian cancer patients, the MLP achieved an accuracy of 70.00% (95% CI: [58.00–80.00]) with a precision of 77.50% (95% CI: [71.05–85.01]), recall of 83.78% (95% CI: [70.27–94.59]), and F1 score of 80.52% (95% CI: [72.00–87.50]) at the time of cancer diagnosis.

Given the sample size, confidence intervals are given to quantify uncertainty in the reported point estimates.

### 3.5. Anecdotal Evidence of SMAART-AI Tool’s Utility

**Figure 5:**
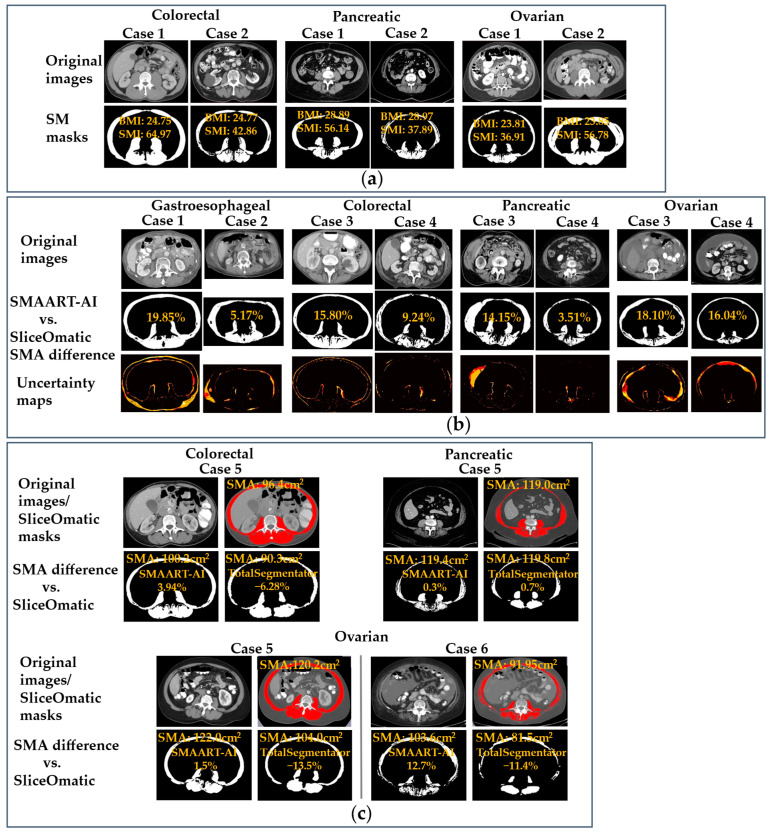
Illustrative examples of SMAART-AI performance to show the effects of patient and image variability). (**a**) The CT images of patients with similar BMIs but different SMIs highlight the added diagnostic value of the SMI in addition to the BMI. In all these examples, despite having similar BMI values, one patient has an SMI that is above the threshold point defined in the literature for sarcopenia/cachexia diagnosis. Another patient has an SMI value below the threshold for cancer cachexia [2,3,10,56,57]. (**b**) Examples of noisy and out-of-distribution scans in which SMAART-AI produced inaccurate skeletal muscle masks. Across all four datasets, the percentage difference in SMA estimated by SMAART-AI is benchmarked against the manual SMA estimated from SliceOmatic. The uncertainty maps visually highlight regions of low model confidence within the muscle segmentation and are displayed using a heat scale from black (lowest uncertainty or highest confidence) to white (highest uncertainty or lowest confidence), with red, orange, and yellow indicating increasing uncertainty, primarily along skeletal muscle boundaries where ensemble predictions disagree. (**c**) Comparison of skeletal muscle masks generated by SMAART-AI, TotalSegmentator, and SliceOmatic at the same manually identified mid-L3 slice. SMAART-AI and TotalSegmentator masks are displayed in white, whereas SliceOmatic skeletal muscle annotations are shown in red. These examples illustrate the tendency of TotalSegmentator to underestimate skeletal muscle. Even in cases where TotalSegmentator SMA estimates are similar to those from SliceOmatic (for example, pancreatic Case 5), the resulting skeletal muscle mask remains anatomically incomplete.

Figure 5a presents samples from the colorectal, pancreatic, and ovarian datasets, featuring two patients from each cancer site with similar BMI but different SMI. In each pair, one patient’s SMI exceeds the literature-defined thresholds for diagnosing sarcopenia/cachexia, while the other’s SMI falls below these thresholds [11,61,62]. In the sample images of the colorectal dataset, both are male patients with BMIs of 24.75 and 24.77, whereas the SMIs are 64.97 and 42.86. The female patients from the pancreatic dataset have BMIs of 28.89 and 28.97, and SMIs of 56.14 and 37.89. The images shown for ovarian cancer patients have BMIs of 23.81 and 23.85, while the SMIs are 36.91 and 56.78.

Figure 5b shows two samples from each dataset, of noisy or out-of-distribution CT images with the corresponding skeletal muscle mask and uncertainty map produced by SMAART-AI. Looking at the gastroesophageal Case 1 and 2 samples, the pixel counts segmented as skeletal muscle (and serving as a proxy for SMA) were 22,404/30,976 using SMAART-AI versus 18,694/29,453 from SliceOmatic. The differences in the pixel counts segmented as skeletal muscle by SMAART-AI compared to the manual segmentation (SliceOmatic) are 19.85% and 5.17%, respectively. In the colorectal Case 3 and 4 samples, the SMAs estimated using SMAART-AI were 79.65 cm^2^/117.76 cm^2^ versus DAFS, which were 49.93 cm^2^/79.85 cm^2^, and 63.48 cm^2^/102.12 cm^2^ using SliceOmatic. The SMAs for pancreatic Case 3 and 4 samples were 151.40 cm^2^/108.40 cm^2^ using SMAART-AI, versus 111.80 cm^2^/100.40 cm^2^ using AW Server, and 132.40 cm^2^/104.70 cm^2^ with SliceOmatic. For the ovarian Case 3 and 4 samples, the SMAs estimated using SMAART-AI were 98.91 cm^2^/138.09 cm^2^ versus 101.00 cm^2^/178.30 cm^2^ from ABACS, and 83.75 cm^2^/119.00 cm^2^ with SliceOmatic. The uncertainty masks visually show areas in the image where the segmentation DL model was underconfident in deciding whether the pixels belonged to skeletal muscle or not.

Figure 5c compares the skeletal muscle masks generated automatically by TotalSegmentator and SMAART-AI and manually using SliceOmatic. These samples illustrate the underestimated skeletal muscle masks generated by TotalSegmentator compared to SMAART-AI and SliceOmatic at the same mid-L3 slice. The colorectal Case 5 sample image shows overestimation by SMAART-AI with an SMA of 100.20 cm^2^ and underestimation by TotalSegmentator with an SMA of 90.30 cm^2^ compared to manual segmentation using SliceOmatic, giving an SMA of 96.40 cm^2^. The SMA estimated by DAFS was 94.10 cm^2^ at the mid-L3 slice, automatically determined by its own internal technique. The pancreatic Case 5 sample image shows close SMA estimation by SMAART-AI of 119.40 cm^2^ and TotalSegmentator of 119.80 cm^2^ compared to manual segmentation using SliceOmatic, with SMA estimation of 119.00 cm^2^. The skeletal muscle mask generated by TotalSegmentator is not complete, but some pixels that do not belong to the skeletal muscle have been marked. Hence, the SMA estimated is close to that of SMAART-AI and SliceOmatic (manual segmentation). AW Server used manual estimation based on Hounsfield windowing at the same mid-L3 slice, to estimate an SMA of 112.30 cm^2^, which is an underestimation compared to the SMA from manual segmentation using SliceOmatic of 119.00 cm^2^. In the ovarian Case 5 image sample, the SMA estimated using SMAART-AI is 122.00 cm^2,^ which is close to the SMA from SliceOmatic, 120.20 cm^2^. However, the SMA estimated using TotalSegmentator is 104.00 cm^2^, which is an underestimation compared to SliceOmatic. ABACS underestimated the SMA to be 114.60 cm^2^ compared to manual segmentation using SliceOmatic at the same mid-L3 slice. The ovarian Case 6 sample image shows overestimation using SMAART-AI, with an SMA of 103.60 cm^2^, and underestimation using TotalSegmentator, with an SMA of 81.50 cm^2^, compared to manual segmentation using SliceOmatic, which gave an SMA of 91.95 cm^2^. ABACS overestimated the SMA to be 114.80 cm^2^ compared to both SMAART-AI and manual segmentation using SliceOmatic at the same mid-L3 slice.

## 4. Discussion

SMAART-AI addresses key barriers that have limited prior tools and demonstrates how imaging-derived biomarkers can be translated into clinically actionable insights through robust segmentation, uncertainty-aware reliability, and multimodal prognostic modeling. A major strength of SMAART-AI is its demonstrated generalizability across heterogeneous datasets, despite being trained primarily on gastroesophageal and pancreatic cancer patients. Validation in colorectal and ovarian cohorts, which differed in imaging protocols, cancer types, and patient body habitus, confirmed robust performance under domain shift. This robustness stems from the use of a structurally diverse ensemble and extensive data augmentation, which together mitigates overfitting and enhance reliability on unseen data. These findings highlight how ensemble diversity and design choices translate into real-world stability, a critical prerequisite for the clinical adoption of AI tools in oncology.

Comparative analysis against widely used proprietary and open-source tools (ABACS, DAFS, AW Server, and TotalSegmentator) underscored the unique advantages of SMAART-AI. While existing methods reduced annotation time, they frequently underestimated the SMA or lacked transparency in outputs, limiting reliability in clinical settings. SMAART-AI achieved performance within the range of inter-expert variability (0.5–1%), with slight overestimation attributable to connective tissue misclassification. Importantly, unlike proprietary solutions, SMAART-AI provides open-source code, model weights, and uncertainty metrics, features essential for reproducibility, interpretability, and clinical trust. These attributes position SMAART-AI not only as accurate but also as a more reliable and transparent alternative for integration into oncology workflows.

Performance degradation under noisy or out-of-distribution conditions was observed across all evaluated tools, reflecting the inherent challenges of real-world clinical imaging. This issue was most evident in the ovarian cohort, which included scans with variable quality and artifacts. Conventional approaches, such as HU thresholding in the AW Server, and even advanced AI-based tools, have proven sensitive to acquisition differences and scanner variability. In contrast, SMAART-AI incorporates uncertainty-aware safeguards that identify cases at higher risk of segmentation error, enabling expert review before results are used in clinical decision-making. This ability to flag unreliable outputs addresses a critical gap in existing tools, enhancing both patient safety and clinician trust in automated pipelines.

Our evaluation of multiple uncertainty estimation strategies (ensemble, dropout, and calibration) confirmed that higher uncertainty strongly correlated with segmentation error, enabling the identification of cases most likely to require expert review. Importantly, this establishes a performance-aware method that differentiates SMAART-AI from conventional segmentation tools. At the same time, our findings revealed limitations in which current techniques occasionally produced low-error cases with high uncertainty or, more critically, confident but incorrect predictions. These observations highlight both the promise and the present limitations of uncertainty quantification, motivating future exploration of training-integrated methods, such as Bayesian neural networks, to reduce overconfidence and further enhance clinical reliability.

A distinctive strength of SMAART-AI is its ability to support longitudinal monitoring of skeletal muscle, providing insights that single time point analyses cannot capture [63]. Automated SMI tracking across follow-up scans revealed dynamic patterns, such as that, in cachectic patients, apparent muscle gain was sometimes attributable to edema, whereas non-cachectic patients displayed variable loss trajectories that may reflect treatment toxicity or early onset of cachexia. These findings underscore the clinical value of continuous, automated monitoring, which can detect subtle or transient changes and enable timely intervention strategies, a capability not feasible with manual annotation.

The integration of SMA and SMI with BMI and clinical variables demonstrated the added value of multimodal prognostic modeling for detecting cachexia, predicting recurrence risk, and predicting survival. Across pancreatic, colorectal, and ovarian cancers, models that included muscle biomarkers consistently outperformed those relying solely on BMI, confirming the unique prognostic utility of radiology-derived features. However, the observed gains should be interpreted cautiously because confidence intervals were broad and often overlapping, and formal statistical comparisons were not significant in the current cohort sizes. Accordingly, these results are best viewed as exploratory evidence of feasibility and potential incremental value, warranting validation in larger, multi-institutional cohorts. Importantly, the intended clinical value of SMAART-AI is not limited to incremental improvements in predictive performance, but also includes the operationalization of reliable, uncertainty-aware muscle quantification that can support case triage, longitudinal monitoring, and downstream multimodal modeling. These findings illustrate how quantitative imaging biomarkers can move beyond descriptive measurements to inform oncology decision-making. At the same time, our results point to opportunities for further refinement through the incorporation of laboratory markers, molecular profiles, and treatment-related variables, underscoring the potential of multimodal integration as a foundation for more personalized cancer care.

Several limitations should be acknowledged. Although SMAART-AI generalized across four independent datasets, training remained concentrated in a subset of cancer types and a limited range of acquisition protocols, populations, and artifact patterns, leaving open questions about performance in broader global populations and external institutions. Future work should include larger externally annotated datasets and prospective validation to better quantify generalizability and calibration across sites. In addition, uncertainty estimation improved reliability but did not eliminate failure modes such as out-of-distribution scans, particularly in the ovarian cohort, and occasional confident but incorrect predictions highlight the need for more advanced approaches to uncertainty modeling.

The predictive models were evaluated in modest cohort sizes, which may limit statistical power. As a result, detailed subtype-specific and clinically stratified downstream analyses were beyond the scope of this study and were not performed. Future validation in larger, multi-institutional cohorts will be critical particularly for well-powered stratified analyses. Another limitation of this retrospective study is that several potentially relevant clinical variables (e.g., alcohol/smoking history, BRI, HbA1c, metabolic panel, and lipid panel) were not consistently available across cohorts and therefore could not be incorporated into the current analyses. Future work will evaluate the added value of these variables in multimodal modeling when harmonized data becomes available.

Several practical sources of performance variability and deployment constraints warrant emphasis. First, heterogeneity in CT acquisition and reconstruction (e.g., scanner vendor/model, reconstruction kernel, slice thickness, field-of-view, and contrast timing) can introduce domain shift, affecting both segmentation accuracy and uncertainty calibration. Although our multi-cohort evaluation includes substantial protocol variability, we did not conduct a comprehensive vendor- and protocol-stratified analysis across all cohorts because DICOM metadata was incomplete or non-uniform. Nevertheless, we consistently observed that scans with pronounced artifacts or degraded image quality tended to have higher uncertainty and larger SMA errors, supporting uncertainty-based triage to identify cases most likely to benefit from review. Second, extreme body habitus may reduce performance via truncation, increased noise, altered anatomy, or partial-volume effects. While SMAART-AI’s triage mechanism is intended to flag atypical appearances, future work should explicitly quantify performance across BMI strata and related measures of body habitus. Finally, deployment in primary hospitals may be constrained by limited IT support for PACS integration, restricted computing resources, greater protocol variability, and fewer expert readers for quality control. These realities motivate staged rollout strategies in which uncertainty-based triage reduces the manual review burden and facilitates gradual integration into routine workflows.

Finally, although the framework is fully automated, prospective evaluation in clinical trials is essential to confirm usability, workflow integration, and real-world impact. Addressing these limitations will be central to advancing SMAART-AI from a validated research tool to a clinically adopted system.

## 5. Conclusions

Cancer cachexia remains a critical driver of poor outcomes, underscoring the need for early detection and continuous monitoring. We introduced SMAART-AI, an open-source, automated, and uncertainty-aware pipeline for skeletal muscle analysis that is robust across multiple cancer types and imaging conditions. By benchmarking against existing tools, we demonstrated both accuracy and transparency while integrating uncertainty estimation to safeguard clinical deployment. Beyond segmentation, SMAART-AI enabled longitudinal tracking of muscle loss and improved prognostic modeling when combined with clinical variables, highlighting the value of radiology-derived biomarkers over BMI alone. Together, these advances establish SMAART-AI as a step toward trustworthy, clinically translatable AI for cachexia management and oncology decision support. Future work will expand integration with additional data modalities and incorporate training-integrated uncertainty modeling to further enhance reliability and clinical impact.

## Figures and Tables

**Table 1:** Datasets used for training and evaluation of the deep learning segmentation, survival, and prediction models.

Cancer Site	Segmentation Models					Survival/Prediction Models	
	No. of Patients	No. of CT Scans	Training Set (Images)	Testing Set (Images)	Annotated (Images)	Training Set (Patients)	Testing Set (Patients)
Gastroesophageal	24	70	45	25	70	-	-
Colorectal	60	60	0	90 *	53	40	20
Pancreatic	153	222 ^†^	15	222 ^	109	100	30
Ovarian	324	324	0	324	154	125	50

Note: The patients used in survival and prediction models are a subset of the patients used in the segmentation models. * One image per axial series at mid-L3 from more than one axial series per CT scan for a subset of patients. ^†^ More than one CT scan per patient, one scan at each time point for a subset of patients. ^ The images used in the training set were a different slice at L3 than the ones used in the testing set.

**Table 2:** Patient characteristics summary for the cohorts used in developing survival and prediction models.

	Colorectal	Ovarian	Pancreatic	
Total patient count	60	175	130	
Age at diagnosis, mean (SD)	61.93 ± 12.50	64.29 ± 10.58	67.81 ± 10.80	
BMI at diagnosis, mean (SD)	27.50 ± 5.84	27.83 ± 6.00	28.18 ± 6.56	
Weight at diagnosis, mean (SD)	172.97 ± 42.99	162.17 ± 35.41	175.77 ± 43.59	
Height at diagnosis, mean (SD)	1.68 ± 0.10	1.63 ± 0.07	1.68 ± 0.11	
Sex, N				
Female	28	175	58	
Male	32	0	72	
Ethnicity, N			Race and Ethnicity, N	
Non-Hispanic/Non-Latinx	53	165	Non-Hispanic White	107
Hispanic/Latinx	7	10	Hispanic/Latinx	13
Race, N			Non-Hispanic Black	10
White	56	163		
Black	0	6		
Other	4	6		
Stage, N	AJCC-7	FIGO	TNM Stage (Pathological), N	
I	8	9	1: 0 (T0/Tis, N0, M0)	8
II	26	14	2: IA (T1, N0, M0)	17
III	24	116	3: IB (T2, N0, M0)	15
IV	1	36	4: IIA (T3, N0, M0	20
NA	1		5: IIA (T1, N1, M0)	1
Grade/Differentiation, N			6: IIA (T2, N1, M0)	8
Well	3		7: IIB (T3, N1, M0)	4
Moderate	41	6	8: III (T4, Any N, M0)	19
Poor	5	45	9: IV (Any T, Any N, M1)	25
Undifferentiated	6	83	99: NA	13
NA	5	41		
Tumor Sequence number *, N				
00		5		
01		23		
02		7		
03		140		

* Tumor sequence number indicates the order of primary tumors recorded for a patient. NA = not available, SD = standard deviation.

**Table 4:** Pearson correlation coefficient between skeletal muscle area (SMA) differences and uncertainty metrics using two uncertainty calibration methods, dropout and ensemble.

Uncertainty Methods and Metrics	Dropout	Ensemble			
	GE	GE	CRC	Pan	Ova
Average Probability	−0.863 *	−0.842 *	−0.487 *	−0.503 *	−0.763 *
Average Calibrated Probability		−0.813 *	−0.442 *	−0.316	−0.782 *
Coefficient of Variation (pixel-wise)	0.739 *	0.852 *	0.529 *	0.526 *	0.756 *
Coefficient of Variation (SMA)	0.296	0.910 *	0.759 *	0.522 *	0.660 *
Average Variance	0.720 *	0.866 *	0.571 *	0.546 *	0.755 *
Average Variance (SM)	0.664 *	0.723 *	0.647 *	0.523 *	0.798 *
Average Entropy	0.867 *	0.843 *	0.474 *	0.516 *	0.749 *
Expected Entropy of the Ensemble	0.869 *	0.701 *	−0.442 *	−0.316	0.655 *

* Significant (*p*-value < 0.05); SM = skeletal muscle; SMA = skeletal muscle area; CRC = colorectal, GE = gastroesophageal, Pan = pancreatic, and Ova = ovarian datasets.

**Table 5:** Triage performance of uncertainty thresholds for detecting high-error SMA estimates (>2.5% vs. SliceOmatic).

Dataset	Uncertainty Metric	Threshold	Flagged%	Threshold Percentile	Sensitivity	Specificity
Gastroesophageal	CoV (SMA)	2.00	20.00%	80.00%	80.00%	95.00%
	Avg variance	0.50	28.00%	72.00%	80.00%	85.00%
Colorectal	Avg variance	0.12	67.90%	32.10%	71.00%	36.40%
		0.20	34.00%	66.00%	38.70%	72.70%
	CoV (SMA)	0.30	67.90%	32.10%	74.20%	40.90%
		0.50	43.40%	56.60%	45.20%	59.10%
Pancreatic	Avg variance	0.20	48.60%	51.40%	65.70%	59.50%
		0.40	14.70%	85.30%	25.00%	90.40%
	CoV (SMA)	0.60	49.50%	50.50%	62.90%	56.80%
		1.00	29.40%	70.60%	42.90%	77.00%
Ovarian	Avg variance	0.35	66.23%	33.77%	79.60%	70.00%
		0.60	36.36%	63.64%	45.60%	89.70%
	CoV (SMA)	1.00	70.78%	29.22%	82.10%	58.50%
		1.50	46.10%	53.90%	57.10%	82.90%

CoV = coefficient of variation.

**Table 6:** Survival analysis showing concordance index with confidence interval (CI) for pancreatic, colorectal, and ovarian datasets.

Dataset	With BMI/SMI/SMA	With SMI/SMA	With BMI	Without BMI/SMI/SMA
Colorectal	0.524 [0.29–0.80]	0.560 [0.34–0.83]	0.500 [0.28–0.78]	0.548 [0.32–0.80]
Pancreatic	0.660 [0.51–0.78]	0.629 [0.48–0.76]	0.613 [0.48–0.73)]	0.601 [0.47–0.71]
Ovarian	0.676 [0.56–0.78]	0.676 [0.54–0.77]	0.659 [0.52–0.77]	0.659 [0.52–0.77]

Note: Combinations of BMI/SMI/SMA are on top of the clinical data that includes patient demographics (sex, race, ethnicity, and age), anthropometric measurements (weight and height), and cancer stage.

## Data Availability

The data supporting this study’s findings is available from Moffitt Cancer Center and the Florida Pancreas Collaborative, but restrictions apply. This data was used under license for the current study and is not publicly available. However, data is available from the authors upon reasonable request and with permission of the Moffitt Cancer Center and the Florida Pancreas Collaborative. The code files for this work are available here: https://github.com/Beemd/SM_Segmentation (accessed on 12 March 2025).

## Supplementary Materials

The following supporting information can be downloaded at: https://www.mdpi.com/article/10.3390/cells15060515/s1, Figure S1: Comparative analysis of SMA estimation using different tools for colorectal cancer; Figure S2: Benchmarking SMA estimation by SMAART-AI versus SliceOmatic for colorectal cancer; Figure S3: Comparative analysis of SMA estimation using different tools for pancreatic cancer; Figure S4: Benchmarking SMA estimation by SMAART-AI and AW Server versus SliceOmatic for pancreatic cancer; Figure S5: Comparative analysis of SMA estimation using different tools for ovarian cancer; Figure S6: Benchmarking SMA estimation by SMAART-AI and ABACS versus SliceOmatic for ovarian cancer; Table S1: Confusion-matrix counts for uncertainty-threshold triage operating points across cohorts; Table S2: Biomarkers and assessment markers reported in the cancer cachexia literature, with relevance to the current study; Table S3: Confirmed diagnosis of pancreatic (n = 130) and Ovarian (n = 175) cohorts; Table S4: List of Abbreviations used in the article; Table S5: Software and Hardware Requirements; DICOM Preprocessing.
