# Supplementary material for: Reliable Radiologic Skeletal Muscle Area Assessment—A Biomarker for Cancer Cachexia Diagnosis
Source: Cells. 2026 Mar 13;15(6):515. doi: 10.3390/cells15060515 (PMC13025493; doi:10.3390/cells15060515)
Supplement: Supplementary file 1 [file cells-15-00515-s001.zip › cells-4161340-supplementary.pdf]

Supplementary Material
Reliable Radiologic Skeletal Muscle Area Assessment – A Biomarker for Cancer Cachexia Diagnosis

DICOM Preprocessing

Preprocessing. The DICOM CT images in the training, validation, and test datasets were adjusted to the skeletal muscle HU range of −29 to 150, followed by conversion to PNG format. The PNG images were normalized by subtracting the mean and dividing by the standard deviation, resulting in pixel values converted to a standard normal distribution with a mean of 0 and a standard deviation of 1.

Postprocessing. The output segmentation masks from the DL segmentation model are converted to PNG and DICOM formats. The DICOM format enables the user to view the generated mask in a DICOM viewer and make corrections if deemed necessary.

The L3 slices identified by the automated pipeline in each patient scan were processed to determine the mid-L3 slice and derive the corresponding skeletal muscle area. The mid-L3 SMA was estimated in two ways:

1. Identifying a single mid-L3 slice based on the following rules:
   - Mid-slice-index = (slice-count/2) – 1, for mid-L3 slice-count ≤ 12
   - Mid-slice-index = (slice-count/2) – 2, for mid-L3 slice-count ≤ 32
   - Mid-slice-index = (slice-count/2) – 3, for mid-L3 slice-count > 32
2. Calculating the average area of mid-L3 and adjacent slices as an alternative to the single mid-L3 SMA:
   - For mid-L3 slice count > 12: The average area was calculated from five slices, two slices above and two slices below the identified mid-L3 slice, including the mid-L3 slice itself.
   - For mid-L3 slice count ≤ 12: The average area was calculated from three slices, one above and one below the identified mid-L3 slice, including the mid-L3 slice itself.

Table S1. Confusion-matrix counts for uncertainty-threshold triage operating points across cohorts.

Cohort	Metric	Threshold	Total (N)	Below threshold	Above threshold	TP	FP	FN	TN
GE	CoV (SMA)	2.00	25	20	5	4	1	1	19
	Avg variance	0.50	25	18	7	4	3	1	17
CRC	Avg variance	0.12	53	17	36	22	14	9	8
		0.20	53	35	18	12	6	19	16
	CoV (SMA)	0.30	53	17	36	23	13	8	9
		0.50	53	30	23	14	9	17	13
Pan	Avg variance	0.20	109	56	53	23	30	12	44
		0.40	109	93	16	9	7	27	66
	CoV (SMA)	0.60	109	55	54	22	32	13	42
		1.00	109	77	32	15	17	20	57
Ova	Avg variance	0.35	153	51	102	90	12	23	28
		0.60	153	97	56	52	4	62	35
	CoV (SMA)	1.00	153	44	109	92	17	20	24
		1.50	153	82	71	64	7	48	34

CoV = coefficient of variation, GE=Gastroesophageal, CRC=Colorectal, Pan=Pancreatic, Ova=Ovarian
Below threshold = not flagged, Above threshold = flagged, TP = high-error flagged, FP = low-error flagged, FN = high-error not flagged, TN = low-error not flagged

Table S2. Biomarkers and assessment markers reported in the cancer cachexia literature, with relevance to the current study.

Category	Biomarker / Marker	Modality / Sample	What it reflects	Typical use in literature	Relevance to this study
Clinical / Anthropometric	Unintentional weight loss (%)	Clinical history / chart review	Core cachexia burden / progression	Diagnosis, staging, monitoring	**Used** (cachexia labeling in pancreatic cohort)
Clinical / Anthropometric	Body mass index (BMI)	Height + weight	Nutritional status / body habitus	Diagnosis support, prognosis, modeling	**Used**
Clinical / Anthropometric	Low BMI threshold (e.g., underweight)	Height + weight	Depletion risk	Diagnosis support (with weight loss/sarcopenia)	**Used indirectly** (via BMI)
Clinical / Anthropometric	Reduced food intake / anorexia	Clinical assessment / PRO	Intake reduction / anorexia domain	Diagnosis/staging support, symptom burden	Not available in current cohorts
Clinical / Anthropometric	Nutrition impact symptoms (e.g., early satiety, nausea, dysphagia)	Clinical assessment	Reduced intake and symptom burden	Supportive assessment / severity	Not available in current cohorts
Clinical / Anthropometric	Performance status (ECOG / WHO)	Clinical assessment	Functional reserve / disease burden	Prognosis, staging support	Not available in current cohorts

Category	Biomarker / Marker	Modality / Sample	What it reflects	Typical use in literature	Relevance to this study
Functional	Handgrip strength (HGS)	Functional test	Muscle strength / functional impairment	Cachexia phenotyping, prognosis, treatment response	Not available
Functional	Gait speed / physical performance tests	Functional test	Functional decline	Supportive assessment / severity	Not available
Functional	Patient-reported physical function / QoL	PRO instruments	Functional impairment / symptom impact	Supportive assessment, longitudinal follow-up	Not available
Imaging / Body Composition	**Skeletal Muscle Area (SMA)** (e.g., L3 CT)	CT imaging	Skeletal muscle mass (cross-sectional)	Diagnosis support, prognosis, longitudinal monitoring	**Primary imaging biomarker (focus of study)**
Imaging / Body Composition	**Skeletal Muscle Index (SMI)** (SMA normalized by height^2)	CT imaging + height	Size-adjusted muscle depletion	Sarcopenia/cachexia assessment, prognosis	**Primary imaging biomarker (focus of study)**
Imaging / Body Composition	Longitudinal change in SMA / SMI	Serial CT imaging	Muscle loss trajectory over time	Monitoring progression / treatment effects	**Used / supported by SMAART-AI longitudinal outputs**
Imaging / Body Composition	Muscle attenuation / radiodensity (HU) (myosteatosis-related)	CT imaging	Muscle quality / fat infiltration	Prognosis, body composition phenotyping	Not used in current models
Imaging / Body Composition	Psoas area / psoas index	CT imaging	Proxy for muscle mass	Simplified sarcopenia/cachexia studies	Not used
Imaging / Body Composition	Visceral adipose tissue area (VAT)	CT imaging	Adipose depletion / redistribution	Body composition phenotyping, prognosis	Not used
Imaging / Body Composition	Subcutaneous adipose tissue area (SAT)	CT imaging	Adipose depletion / redistribution	Body composition phenotyping, prognosis	Not used
Imaging / Body Composition	Intermuscular adipose tissue / fat infiltration	CT imaging	Muscle quality and ectopic fat	Phenotyping / prognosis	Not used
Laboratory / Inflammatory	C-reactive protein (CRP)	Blood	Systemic inflammation	Diagnosis support, prognosis, severity assessment	Not available in current cohorts
Laboratory / Inflammatory	Albumin	Blood	Nutrition/inflammation status	Prognosis, cachexia assessment support	Not available in current cohorts

Category	Biomarker / Marker	Modality / Sample	What it reflects	Typical use in literature	Relevance to this study
Laboratory / Inflammatory	mGPS (CRP + albumin)	Blood composite score	Inflammatory / nutritional risk	Prognosis, cachexia severity support	Not available
Laboratory / Inflammatory	Prealbumin	Blood	Nutritional status (shorter-term)	Supportive nutritional assessment	Not available
Laboratory / Inflammatory	CRP/albumin ratio	Blood	Inflammation + nutrition composite	Prognostic biomarker (study-dependent)	Not available
Laboratory / Inflammatory	Hemoglobin (Hb)	Blood	Anemia / systemic illness burden	Prognostic and supportive marker	Not available
Laboratory / Inflammatory	WBC / neutrophils / lymphocytes	Blood (CBC)	Inflammatory / immune status	Prognostic and inflammatory profiling	Not available
Laboratory / Inflammatory	NLR / PLR / SII	Derived CBC indices	Systemic inflammation	Prognostic / inflammatory biomarker studies	Not available
Cytokines / Soluble Factors (Investigational)	IL-6	Blood	Pro-inflammatory signaling	Biomarker studies, mechanism-linked analyses	Not available
Cytokines / Soluble Factors (Investigational)	TNF-α	Blood	Pro-inflammatory / catabolic signaling	Biomarker studies	Not available
Cytokines / Soluble Factors (Investigational)	IL-1β / IL-8 / MCP-1 (CCL2)	Blood	Inflammatory mediators	Exploratory biomarker studies	Not available
Endocrine / Metabolic (Investigational)	GDF15	Blood	Appetite/metabolic dysregulation (candidate)	Emerging biomarker studies	Not available
Endocrine / Metabolic (Investigational)	Activin A / Myostatin	Blood	Muscle catabolism signaling (candidate)	Emerging biomarker studies	Not available
Endocrine / Metabolic (Investigational)	Ghrelin / Leptin / Adiponectin	Blood	Appetite and energy homeostasis	Exploratory biomarker studies	Not available
Endocrine / Metabolic (Investigational)	IGF-1 / FGF21 / ZAG (study-dependent)	Blood	Metabolic and catabolic pathways	Exploratory biomarker studies	Not available
Molecular / Omics (Emerging)	Circulating microRNAs (miRNAs)	Blood / plasma / exosomes	Regulatory and pathway signatures	Exploratory diagnostics / phenotyping	Not available
Molecular / Omics (Emerging)	Proteomics / metabolomics / lipidomics signatures	Blood / tissue	Multi-pathway systemic alterations	Biomarker discovery / trial enrichment	Not available
Molecular / Omics (Emerging)	Exosomal biomarkers	Blood	Intercellular signaling cargo	Emerging biomarker discovery	Not available

Table S3. Confirmed diagnosis of pancreatic (n=130) and Ovarian (n=175) cohorts.

**Diagnosis (Pancreatic)**	**Count of patients**
PDAC	87
PNET	17
IPMN	13
pancreas related	3
other	7
Liver/Bile duct/Lymph node/Ampulla related	2
MCN	1
**Histology (Ovarian)**	**Count of patients**
CARCINOMA UNDIFFERENTIATED NOS	1
PAPILLARY CARCINOMA NOS	1
PAPILLARY SEROUS CYSTADENOCARCI	14
SEROUS CYSTADENOCARCINOMA NOS	150
SEROUS SURFACE PAPILLARY CARCIN	6
Not available	3

Table S4. List of Abbreviations used in the article.

A. Core study / imaging / modeling abbreviations

**Abbreviation**	**Expansion**
**CT**	Computed tomography
**SMA**	Skeletal muscle area
**SMI**	Skeletal muscle index
**SMAART-AI**	Skeletal Muscle Assessment-Automated and Reliable Tool based on AI
**AI**	Artificial intelligence
**BMI**	Body mass index
**BIA**	Bioelectrical impedance analysis
**DL**	Deep learning
**CNNs**	Convolutional neural networks
**L3**	Third lumbar vertebral level
**L4**	Fourth lumbar vertebral level
**HU**	Hounsfield unit
**MLP**	Multilayer perceptron
**LoA**	Limits of agreement
**C-index**	Concordance index
**CI**	Confidence interval

B. Tool / software / pipeline abbreviations

Abbreviation	Expansion
**ABACS**	Automatic Body Composition Analyzer using Computed Tomography Image Segmentation
**DAFS**	Data Analysis Facilitation Suite
**AW Server**	Advanced Workstation Server
**nnU-Net**	(Model/framework name)
**U-Net**	(Model architecture name)
**TotalSegmentator**	(Tool name)
**netcal**	(Library name)
**DICOM**	Digital Imaging and Communications in Medicine
**PACS**	Picture Archiving and Communication System
**VNA**	Vendor Neutral Archive
**PNG**	Portable Network Graphics
**NIfTI**	Neuroimaging Informatics Technology Initiative (format)
**ReLU**	Rectified Linear Unit

C. Clinical / disease / cohort abbreviations

Abbreviation	Expansion
**IPMN / IPMNs**	Intraductal papillary mucinous neoplasm(s)
**PDAC**	Pancreatic ductal adenocarcinoma
**PNET**	Pancreatic neuroendocrine tumor
**TTE**	Time to event
**GE**	Gastroesophageal
**CRC**	Colorectal
**Pan**	Pancreatic
**Ova**	Ovarian
**SM**	Skeletal muscle

D. Table-only / staging / stats abbreviations (important reviewer-facing items)

Abbreviation	Expansion
**SD**	Standard deviation
**N**	Number (count)
**AJCC-7**	American Joint Committee on Cancer, 7th edition
**FIGO**	International Federation of Gynecology and Obstetrics
**TNM**	Tumor, Node, Metastasis
**NA**	Not available / Not applicable
**CoV**	Coefficient of variation
**TP / FP / FN / TN**	True positive / False positive / False negative / True negative

E. Funding / affiliations / front matter abbreviations (optional to include in abbreviation list, but okay if reviewer is strict)

Abbreviation	Expansion
**CC BY**	Creative Commons Attribution
**GI**	Gastrointestinal
**NCI**	National Cancer Institute
**NIH**	National Institutes of Health
**NSF**	National Science Foundation
**IRB**	Institutional Review Board
**MCC**	(Moffitt protocol IDs)

Table S5. Software and Hardware Requirements.

A. Software Requirements

Category	Software / Framework	Version (Recommended)
Programming Language	Python	3.9 – 3.10
Deep Learning Framework	PyTorch	≥ 2.0
Medical Segmentation Framework	nnU-Net	v2.x
Anatomical Structure Segmentation	TotalSegmentator	≥ 1.5
Uncertainty Calibration	netcal	≥ 1.3
Survival Analysis	lifelines	≥ 0.27
Medical Image Processing	SimpleITK	≥ 2.2
DICOM Handling	pydicom	≥ 2.4
Neuroimaging Format Support	NiBabel	≥ 5.0
Numerical Computing	NumPy	≥ 1.24
Data Processing	pandas	≥ 1.5
Machine Learning Utilities	scikit-learn	≥ 1.2
Visualization	matplotlib	≥ 3.7
Image Operations	OpenCV	≥ 4.7
GPU Acceleration	CUDA	11.7 – 11.8

OS Environment	Ubuntu Linux	20.04 / 22.04 LTS

B. Hardware Requirements

**Component**	**Recommended Specification**	**Role in Pipeline**
GPU	NVIDIA RTX 3090 / A100 / V100 (≥24 GB VRAM)	nnU-Net training, ensemble inference
CPU	≥16 cores (Intel Xeon / AMD EPYC)	Data loading, preprocessing
System RAM	≥64 GB	Multi-series CT handling
Storage	≥1 TB SSD (NVMe preferred)	DICOM + intermediate outputs
GPU Memory	≥24 GB VRAM	Ensemble + uncertainty inference
Network	High-speed institutional network	PACS/VNA CT retrieval
Compute Environment	HPC Cluster or Workstation	Large cohort processing

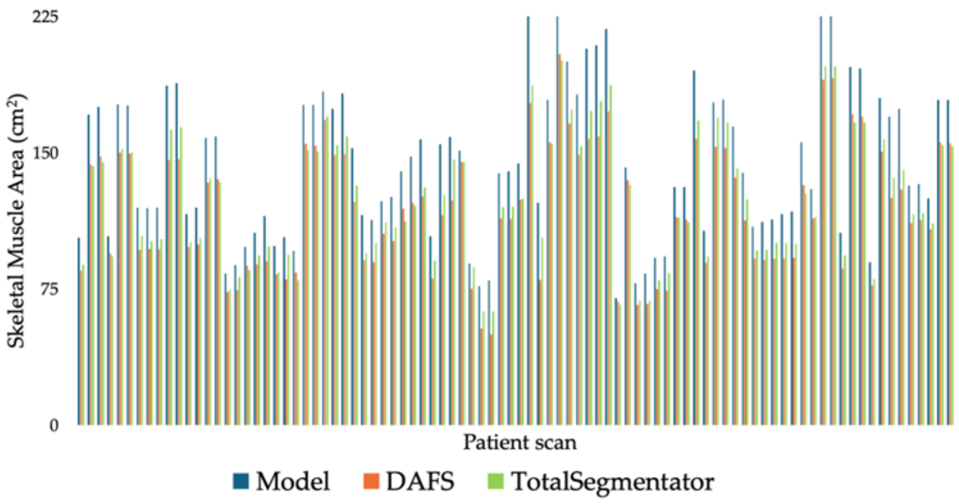

(a)

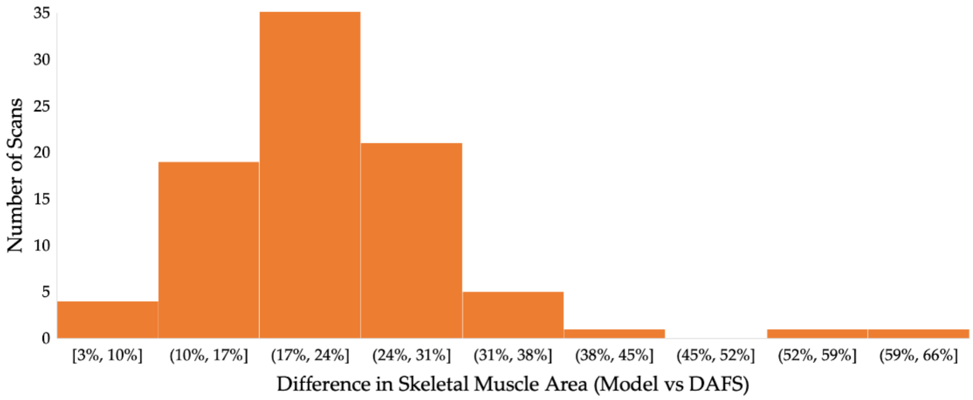

(**b**)

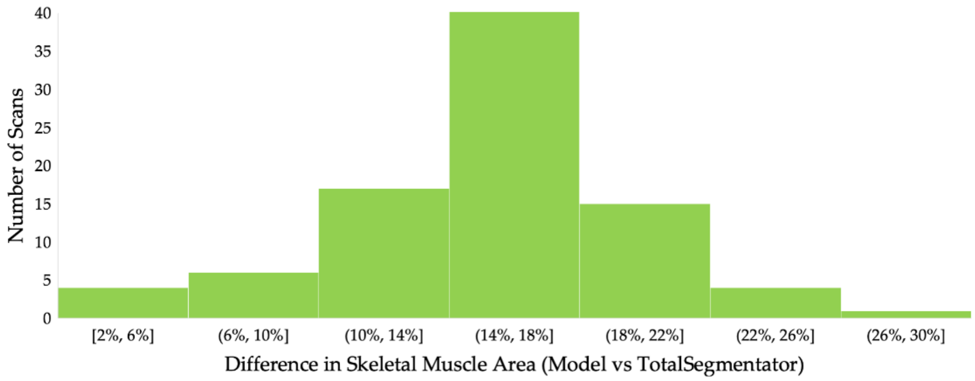

(**c**)

Figure S1. Comparative analysis of SMA estimation using different tools for colorectal cancer. (**a**) Comparison of SMA estimated for 60 patients (90 scans, including multiple axial series per patient) at the mid-L3 level by SMAART-AI, DAFS, and TotalSegmentator. Both DAFS and TotalSegmentator consistently estimate lower SMA values compared to SMAART-AI, with DAFS generally estimating lower values than TotalSegmentator. The mid-L3 slice used by SMAART-AI and DAFS is determined automatically by their respective pipelines, while TotalSegmentator uses the mid-L3 slice determined by our proposed pipeline. (**b**) Distribution of differences between SMA predictions by SMAART-AI and DAFS indicates a large discrepancy, potentially due to variation in the selected mid-L3 slice or poor DAFS performance on this dataset. (**c**) Distribution of differences between SMA predictions by SMAART-AI and TotalSegmentator suggests that TotalSegmentator consistently underestimates SMA in most cases.

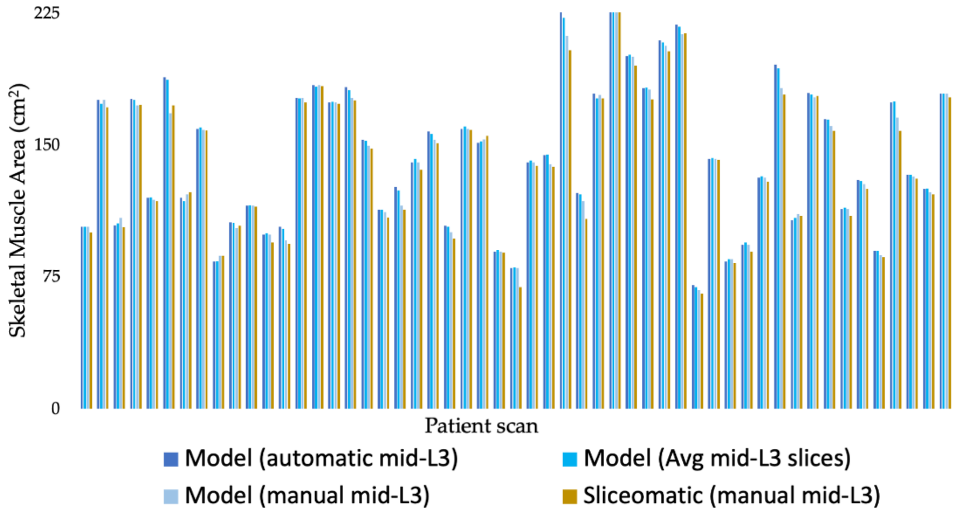

(**a**)

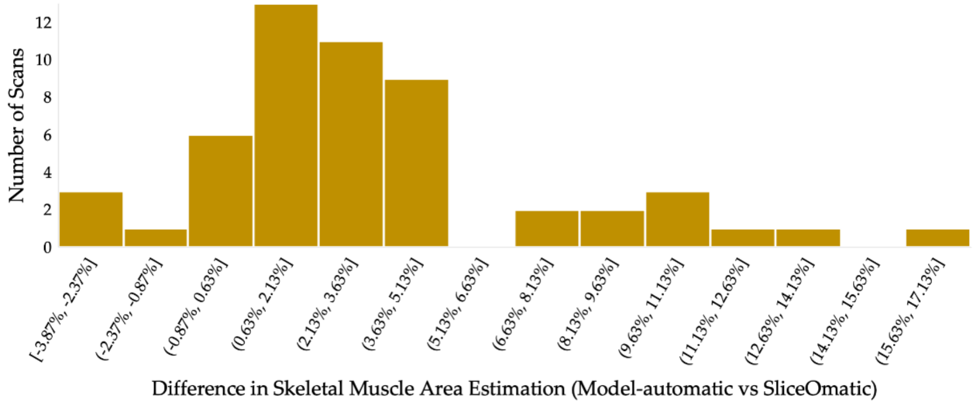

(**b**)

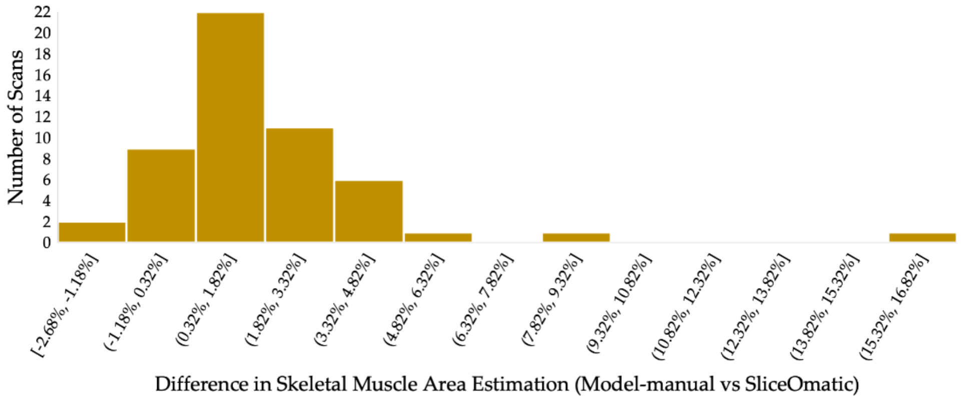

(**c**)

Figure S2. Benchmarking SMA estimation by SMAART-AI versus SliceOmatic for colorectal cancer. (**a**) Comparison of SMA estimates for 53 patients by different methods: SMAART-AI at mid-L3 (automated pipeline), the average of slices around the pipeline determined mid-L3, the model's prediction at the manually determined mid-L3 slice, and SliceOmatic (manual). The SMA values for the manual and automated mid-L3 slices, as well as the average of surrounding slices, are nearly identical in most cases, closely aligning with the estimations from SliceOmatic. (**b**) The distribution of differences between SMAART_AI's SMA estimates (mid-L3

determined by the pipeline) and SliceOmatic (manual) shows strong agreement. Differences greater than 3% are observed when the automated and manually selected mid-L3 slices differ significantly or when the CT image is noisy or out-of-distribution. (**c**) The difference between SMAART-AI's estimation and SliceOmatic at the manually determined mid-L3 indicates strong model performance overall. Larger discrepancies are mainly observed in low-quality images, while differences up to 2% may be attributed to the fact that the model may include connective tissues as part of skeletal muscle. The comparison of the average area of slices near mid-L3 with a single mid-L3 slice has a median difference of 0.53% and a mean difference of 0.66%, indicating that adjacent slices provide similar area estimates. Overall, SMAART-AI outperforms both DAFS and TotalSegmentator.

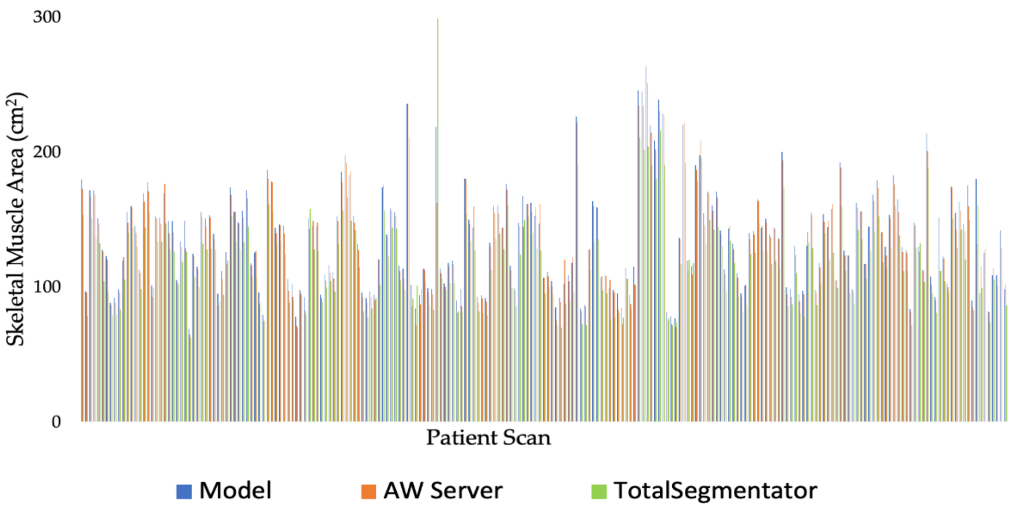

(**a**)

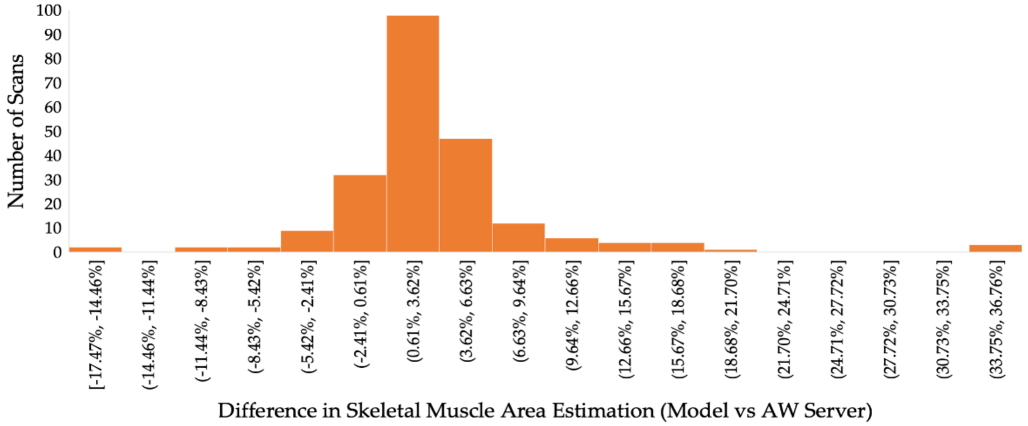

(**b**)

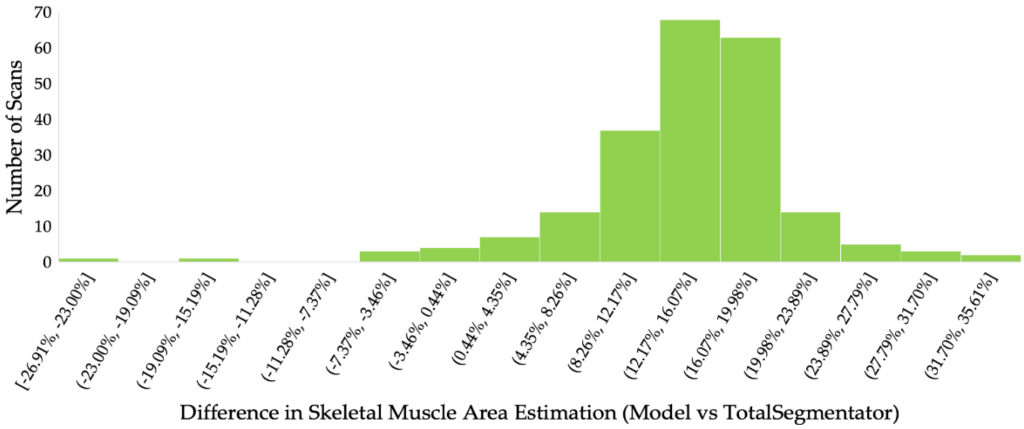

(**c**)

Figure S3. Comparative analysis of SMA estimation using different tools for pancreatic cancer. (**a**) Comparison of skeletal muscle area (SMA) estimates from 222 patient scans at the manually determined end-L3 level by SMAART-AI, AW Server, and TotalSegmentator shows that TotalSegmentator consistently estimates lower values in most cases compared to both SMAART-AI and AW Server. (**b**) The distribution of differences between the SMA estimated by SMAART-AI and AW Server shows an approximately 3% absolute difference in around 60% of cases. Overall, SMAART-AI tends to slightly underestimate in some cases but generally overestimates compared to AW Server. (**c**) The distribution of differences between SMAART-AI and TotalSegmentator indicates significant disagreement in most cases.

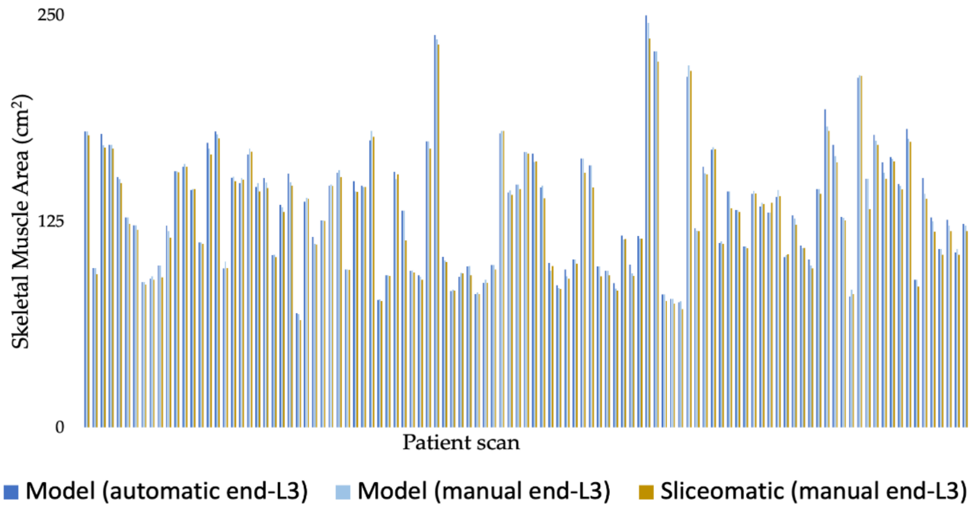

(**a**)

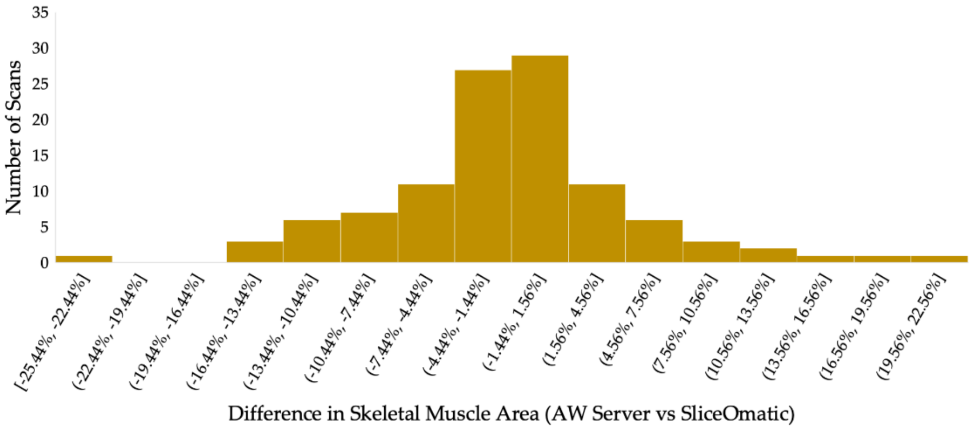

(**b**)

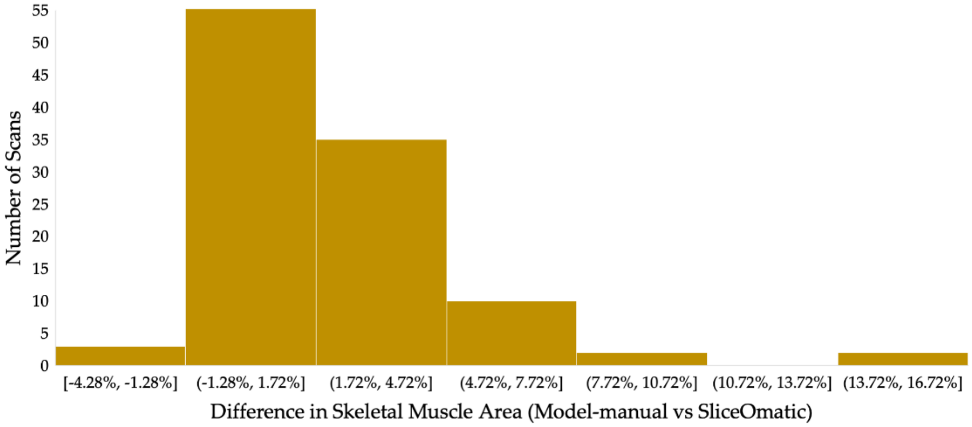

(**c**)

Figure S4. Benchmarking SMA estimation by SMAART-AI and AW server versus SliceOmatic for pancreatic cancer. (**a**) Comparison of SMA estimates for 109 patient scans, using SMAART-AI at both automatically and manually identified end-L3 slices, along with SliceOmatic (at the same manually selected end-L3 slices), shows that the SMA values from both automated and manually selected slices are nearly identical in most cases, closely matching the reference end-L3 SMA determined by SliceOmatic. (**b**) The distribution of differences in SMA between estimates from AW Server and SliceOmatic at the manually determined end-L3 slice shows good agreement in approximately 61% of cases. (**c**) The distribution of differences in SMA between SMAART-AI's estimate at the manually determined end-L3 and SliceOmatic reveals good agreement in around 87% of cases. SMAART-AI tends to overestimate the SMA in some cases, whereas AW Server shows a slight bias towards underestimation. Overall, SMAART-AI performs well.

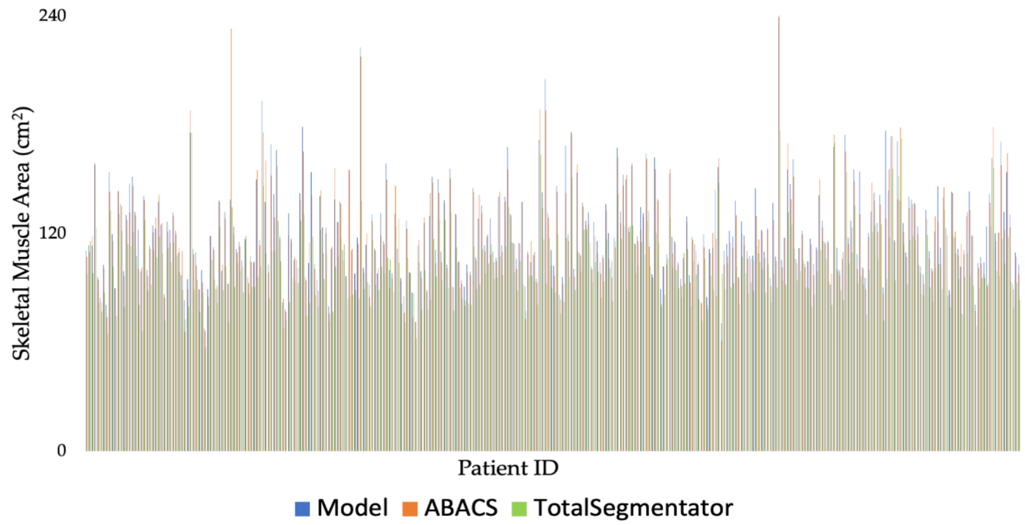

(**a**)

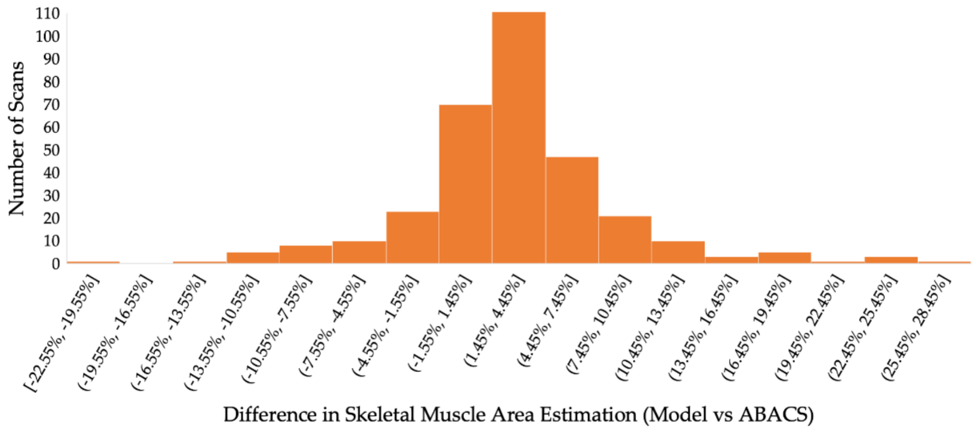

(**b**)

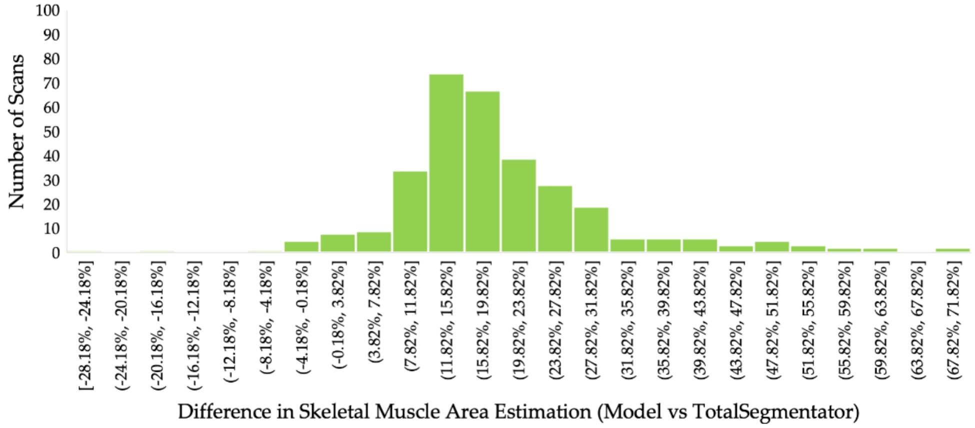

(**c**)

Figure S5. Comparative analysis of SMA estimation using different tools for ovarian cancer. (**a**) A comparison of skeletal muscle area (SMA) from 324 patient scans at the manually determined mid-L3 level, estimated by SMAART-AI, ABACS, and TotalSegmentator, shows that TotalSegmentator estimates lower values compared to the trained model and ABACS with some exceptions. There are a few odd cases where ABACS is estimating very large values for the area compared to SMAART-AI and TotalSegmentator. (**b**) The distribution of the difference between the SMA estimated

by the SMAART-AI and ABACS indicates close agreement in around 63% of cases. (**c**) The distribution of the difference between SMAART-AI and TotalSegmentator shows high disagreement in most cases and a close estimation in only around 4% of the cases.

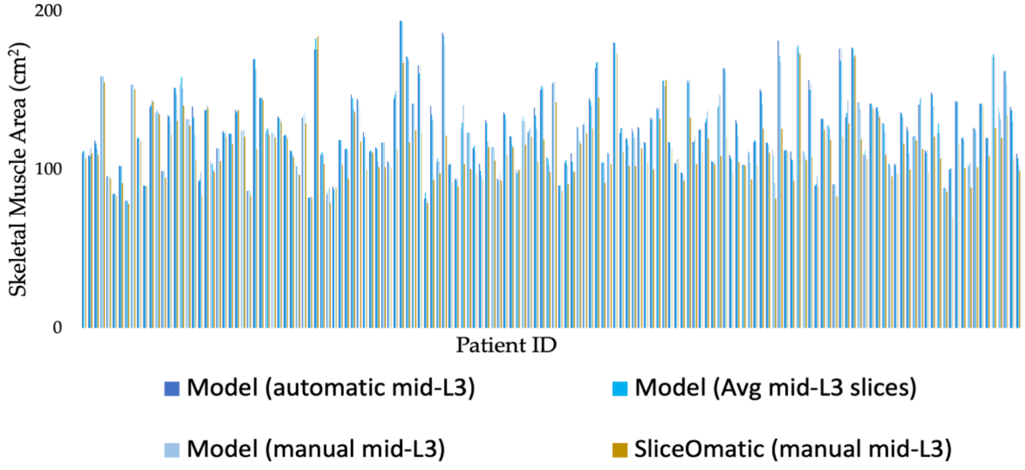

(**a**)

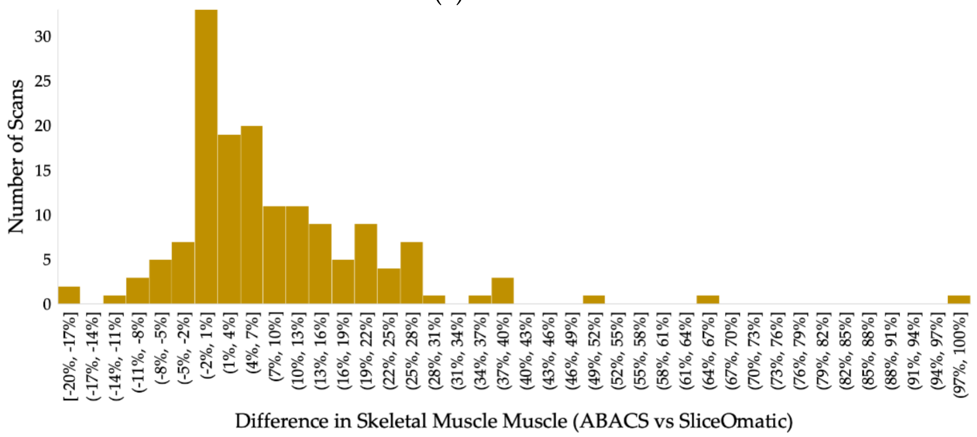

(**b**)

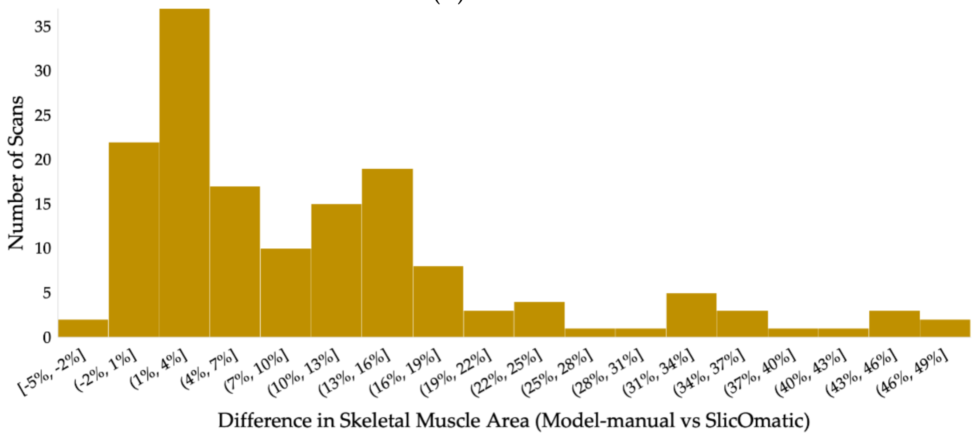

(**c**)

Figure S6. Benchmarking SMA estimation by SMAART-AI and ABACS versus SliceOmatic for ovarian cancer. (**a**) Comparison of SMA estimates from 154 patients' CT scans using different methods: SMAART-AI at mid-L3 (automated), the average of slices around SMAART-AI determined mid-L3, SMAART-AI at the manually

determined mid-L3, and SliceOmatic (manual). In most cases, SMA estimates for mid-L3 (both manual and automatic), and the average of adjacent slices closely match the reference SMA determined by SliceOmatic. (**b**) The distribution of differences between SMA estimated by ABACS and SliceOmatic at the manually determined mid-L3 shows that ABACS provides estimates with low differences in approximately 32% of the cases, with a trend of overestimations and occasional underestimations. (**c**) The distribution of differences between SMA estimated by SMAART-AI at the manually determined mid-L3 and SliceOmatic shows estimation with low difference in around 26% of cases but tends to overestimate in most cases. Overestimations by both ABACS and SMAART-AI are primarily attributed to out-of-distribution or noisy images, which are prevalent in this dataset.
